# Supplementary material for: A Transcriptome-wide Atlas of RNP Composition Reveals Diverse Classes of mRNAs and lncRNAs
Source: Cell. 2013 Aug 29;154(5):996–1009. doi: 10.1016/j.cell.2013.07.047 (PMC3778888; doi:10.1016/j.cell.2013.07.047)
Supplement: Document S1. Tables S1 and S2 [file mmc1.pdf]

Table S1. Summary of Sequencing Datasets, Related to Figure 1

HTP-tagged yeast strain	Total reads after duplicates collapsed	Mapped reads	Mapped reads after second collapsing step	Reads spanning Intron-Exon or Exon-Exon junctions	Hits in Pol II transcribed RNAs
Untagged	132,540	104,828	NA	Not analysed	47619
	3,279	1,945	NA	Not analysed	343
	177,394	105,339	38,377	Not analysed	19801
	64,648	49,122	13,866	Not analysed	6599
Hek2	1,203,569	684,274	365,358	1318	326077
Gbp2	3,384,410	1,747,127	1,006,460	7375	730902
Hrp1	681,331	573,431	NA	1663	345972
	819,092	577,177	NA	2257	351900
Mex67	280,324	231,026	NA	336	114809
	311,754	247,326	NA	323	121804
	530,155	346,093	177,812	481	82351
Mtr4	1,005,308	730,591	208,427	1013	63931
	2,035,764	1,514,999	393,099	2407	142513
	3,783,833	2,591,016	988,042	3971	453470
Nab2	449,579	372,485	285,723	1866	200446
	6,479,021	4,823,691	2,340,092	22455	1715833
	1,452,862	1,058,178	475,551	8106	319860
Nab2 *Δrrp6*	3,413,860	2,307,904	1,307,646	10283	943578
Pab1	31,828,181	24,433,808	6,299,244	25820	5233431
Ski2	1,016,310	819,307	213,604	1604	120541
	1,351,395	1,044,648	674,270	2652	485147
	342,397	247,038	150,355	517	87686
	427,802	307,694	179,033	655	111144
Cbc1	1,436,824	646,108	316,953	5267	242804
	1,056,734	618,448	498,142	5687	403414
	364,865	286,451	197,623	2117	137040
Tho2	744,315	543,570	149,018	1427	83855
Tif1	201,342	116,724	NA	1531	73963
Trf4	313,153	247,673	NA	336	91761
Xrn1	518,797	406,833	NA	1061	288562
	343,514	249,924	NA	623	175192

For all libraries, identical reads were collapsed to remove PCR duplicates (“Total reads after duplicates collapsed”). For libraries with 5’ adapters containing a random 3 nt barcode, additional duplicates were removed post-mapping, by collapsing reads with the same 3 nt barcode and 5’ genomic coordinate. For libraries constructed with 5’ adapters that do not contain the random 3 nt barcode, this second collapsing step could not be performed (indicated by “NA”). “Reads spanning Intron-Exon or Exon-Exon junctions” indicates the subset of reads used for analysis in Figure 1B. “Hits in Pol II transcribed RNAs” indicates the total number of reads that map to mRNAs, CUTs, SUTs, snRNAs and snoRNAs, and this value was used for internal normalization of datasets in Figure 1.

Table S2. Number of mRNAs, CUTs, and SUTs in Each Cluster, Related to Figure 2

Cluster	mRNAs			CUTs		SUTs	
	Count	% of all mRNAs	Intron-containing pre-mRNAs	Count	% of all CUTs	Count	% of all SUTs
I	100	2.5	0	310	55.4	69	15.9
II	190	4.8	5	76	13.6	85	19.6
III	121	3.1	1	141	25.2	131	30.2
IV	441	11.1	12	11	2.0	51	11.8
V	219	5.5	66	10	1.8	42	9.7
VI	532	13.4	55	0	0.0	8	1.8
VII	498	12.6	7	2	0.4	17	3.9
VIII	695	17.5	12	1	0.2	15	3.5
IX	590	14.9	23	5	0.9	9	2.1
X	580	14.6	32	4	0.7	7	1.6

We also include the number of intron-containing (pre-)mRNAs in each cluster.
