# Supplementary material for: A Transcriptome-wide Atlas of RNP Composition Reveals Diverse Classes of mRNAs and lncRNAs
Source: Cell. 2013 Aug 29;154(5):996–1009. doi: 10.1016/j.cell.2013.07.047 (PMC3778888; doi:10.1016/j.cell.2013.07.047)
Supplement: Document S2. Article plus Supplemental Information [file mmc5.pdf]

Cell

A Transcriptome-wide Atlas of RNP Composition Reveals Diverse Classes of mRNAs and lncRNAs

Alex Charles Tuck[1] and David Tollervey[1,*]
[1]The University of Edinburgh, Wellcome Trust Centre for Cell Biology, Michael Swann Building, Kings Buildings, Edinburgh EH9 3JR, UK
*Correspondence: d.tollervey@ed.ac.uk
http://dx.doi.org/10.1016/j.cell.2013.07.047

SUMMARY

Eukaryotic genomes generate a heterogeneous ensemble of mRNAs and long noncoding RNAs (lncRNAs). LncRNAs and mRNAs are both transcribed by Pol II and acquire 5′ caps and poly(A) tails, but only mRNAs are translated into proteins. To address how these classes are distinguished, we identified the transcriptome-wide targets of 13 RNA processing, export, and turnover factors in budding yeast. Comparing the maturation pathways of mRNAs and lncRNAs revealed that transcript fate is largely determined during 3′ end formation. Most lncRNAs are targeted for nuclear RNA surveillance, but a subset with 3′ cleavage and polyadenylation features resembling the mRNA consensus can be exported to the cytoplasm. The Hrp1 and Nab2 proteins act at this decision point, with dual roles in mRNA cleavage/polyadenylation and lncRNA surveillance. Our data also reveal the dynamic and heterogeneous nature of mRNA maturation, and highlight a subset of "lncRNA-like" mRNAs regulated by the nuclear surveillance machinery.

INTRODUCTION

High-throughput transcriptome analyses in eukaryotes have revealed pervasive transcription at most, if not all, genomic loci. This generates many long noncoding RNAs (lncRNAs), which lack protein-coding capacity and are distinct from well-characterized structural RNAs (rRNAs, tRNAs, snRNAs, and snoRNAs) or small regulatory RNAs. LncRNAs arise from intergenic, antisense, or promoter-proximal regions and range in size from ~200 nt to >20 kb. Many features are shared between lncRNAs and mRNAs; both classes of RNA possess 5′-methylguanosine caps (Neil et al., 2009) and poly(A) tails (David et al., 2006) and have broadly similar lengths. Moreover, both are transcribed by RNA polymerase II (Pol II) from similar preinitiation complex assemblies (Rhee and Pugh, 2012) and can be regulated by common transcription factors. Despite these similarities, the fates and functions of lncRNAs and mRNAs are substantially different. Most mRNAs are rapidly exported to the cytoplasm, where they engage with the protein synthesis machinery. In contrast, diverse nuclear functions have been attributed to lncRNAs, including the assembly of nuclear domains, directing chromatin-modification, resetting of epigenetic marks, and the regulation of mRNA transcription.

Several studies have identified classes of lncRNA with distinct features. For example, stable unannotated transcripts (SUTs) are detectable in wild-type yeast, whereas cryptic unstable transcripts (CUTs) are apparent only in the absence of the nuclear surveillance factor Rrp6, and Xrn1-sensitive unstable transcripts (XUTs) are apparent only in the absence of the cytoplasmic exoribonuclease Xrn1 (van Dijk et al., 2011; Xu et al., 2009). This indicated that distinct classes of lncRNAs can be distinguished from each other, as well as from mRNAs, but the features that might differentiate these species were unclear.

All mRNAs interact with a defined series of protein factors during their transcription, packaging, processing, export, and turnover (see Figure 1A), forming ribonucleoprotein particles (RNPs). We hypothesized that lncRNAs and mRNAs must diverge at some point along this maturation pathway. We therefore systematically analyzed the in vivo, transcriptome-wide targets of key factors in this pathway in budding yeast (Figure 1A). We anticipated that this atlas of RNP compositions would provide a comprehensive picture of the dynamic events during canonical messenger RNP (mRNP) assembly, and give insights into the definition and behavior of different classes of mRNAs and lncRNAs.

Overall, our data reveal how distinct transcript classes are defined and how RNP composition relates to function, and have enabled us to begin to tackle the overwhelming complexity of the transcriptome.

RESULTS

Transcriptome-wide Analysis of RNP Composition

To establish how and when different classes of Pol II transcripts are distinguished in the cell, we determined the transcriptome-wide targets for 13 key mRNA biogenesis and turnover factors (listed in Figure 1A and Table 1, with references therein). We included nuclear surveillance factors (Mtr4 and Trf4), a component of the nuclear cap-binding complex (Cbc1), components of the TREX RNA packaging complex (Gbp2 and Tho2),

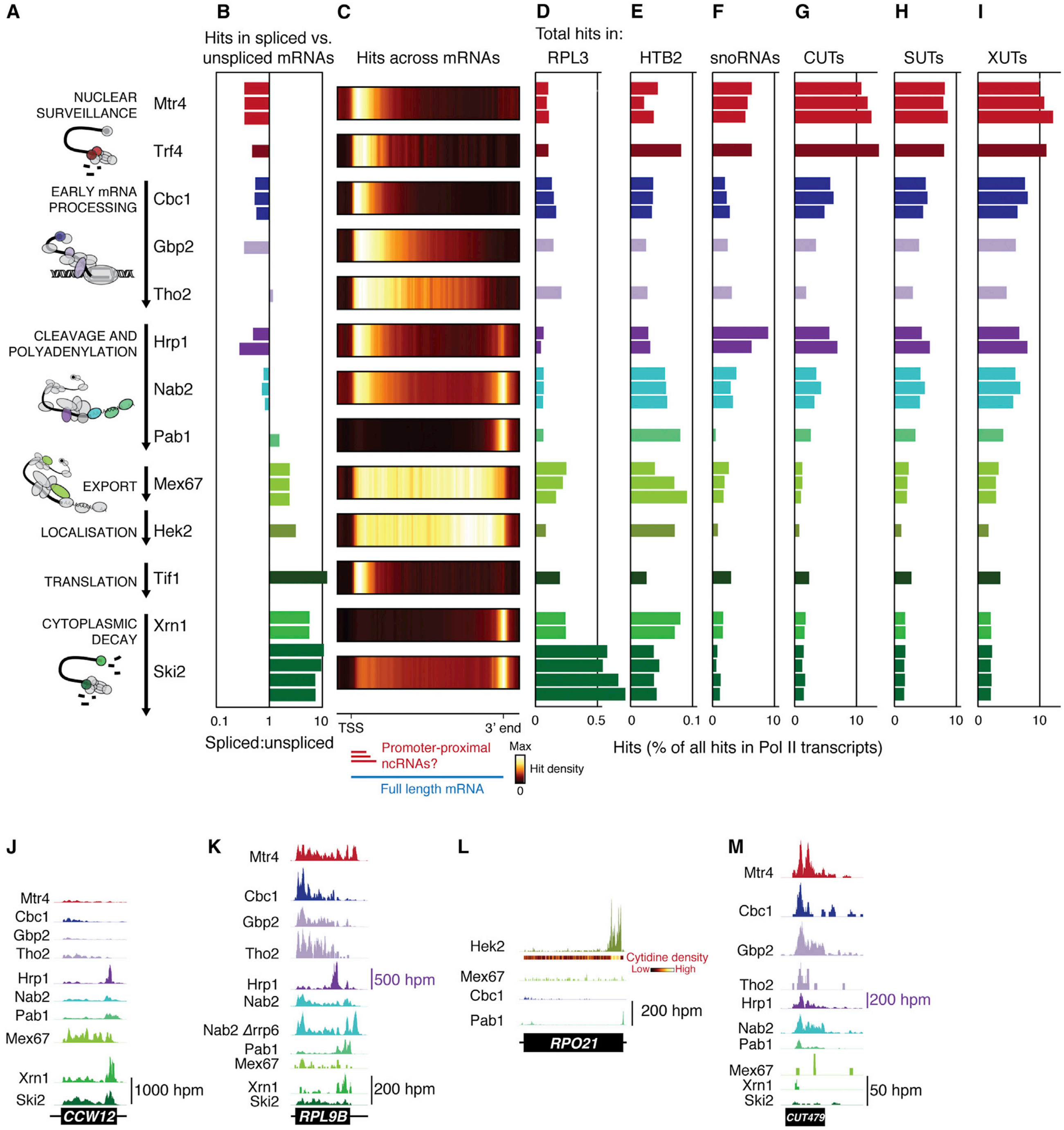

Figure 1. Transcriptome-wide Analysis of RNP Composition

(A) mRNA maturation and decay factors selected for analysis.

(B) Relative recovery of spliced mRNAs versus unspliced pre-mRNAs bound to the tested proteins, expressed as the ratio of RNA fragments spanning exon-exon:intron-exon junctions.

(C) Average binding distribution of the tested proteins across mRNAs. For each protein, average hit densities were calculated for 120 bins spanning their 1,000 most abundantly bound mRNAs (including 2 × 10 bins for 100 nt 5′ and 3′ flanking regions). 5′-proximal hits can arise from binding to promoter-proximal ncRNAs or to the 5′ end of full-length mRNAs.

(D–I) Total hits for each protein in *RPL3* and *HTB2* pre-mRNAs, snoRNAs, CUTs, SUTs, and XUTs, as a percentage of all hits in Pol II transcribed RNAs (mRNAs, CUTs, SUTs, snRNAs, and snoRNAs; Table S1).

(J–M) Hit distributions along individual transcripts, at the indicated scales (hits per million hits in Pol II transcribed RNAs). Note the different scale used for Hrp1 data in K and M due to the high level of binding. See also Figure S1 and Table S1.

Table 1. Proteins Selected for Analysis

Yeast Protein	Human Homolog	Function	References
Nuclear Surveillance			
Mtr4	hMtr4/SKIV2L2	RNA helicase (Mtr4) and noncanonical poly(A) polymerase (Trf4) within the TRAMP complex; assist the nuclear exosome in RNA degradation	Reviewed in Porrua and Libri, 2013
Trf4	hTRF4-1/POLS		
Early mRNP Biogenesis			
Cbc1/Sto1	CBP80	Nuclear cap binding complex subunit; mRNA stabilization, processing, export and decay	Görnemann et al., 2005; Wong et al., 2007
Tho2	Thoc2	Components of the TREX complex; transcription elongation and mRNA export	Reviewed in Rondón et al., 2010
Gbp2			
Cleavage and Polyadenylation			
Hrp1	TDP-43	Cleavage and polyadenylation factor	Kessler et al., 1997
Nab2	ZC3H14	Nuclear poly(A)-binding protein; poly(A) tail length control, mRNA export and nuclear surveillance of pre-mRNAs	Iglesias et al., 2010; Schmid et al., 2012; Viphakone et al., 2008
Pab1	PABPC1	Poly(A)-binding protein; mRNA export, translation and stability	Reviewed in Parker, 2012
Export and Translation			
Mex67	NXF1/Tap	mRNA export receptor	Hieronymus and Silver, 2003
Hek2/Khd1	hnRNP K, hnRNP E and poly(C)-binding proteins	mRNA localization, translational inhibition and stability	Hasegawa et al., 2008; Irie et al., 2002; Mauchi et al., 2010; Paquin and Chartrand, 2008; Vogel et al., 2011; Wolf et al., 2010
Tif1/eIF4A	EIF4A	Helicase within the cytoplasmic cap-binding complex; ribosome scanning of the 5′ UTR	
Cytoplasmic Decay			
Ski2	SKIV2L	Helicase that assists the cytoplasmic exosome in 3′–5′ mRNA turnover	Reviewed in Parker, 2012
Xrn1	XRN1	5′ to 3′ exonuclease in the major cytoplasmic mRNA decay pathway	Reviewed in Parker, 2012

pre-mRNA 3′ cleavage and polyadenylation factors (Hrp1, Nab2, and Pab1), a nuclear-cytoplasmic export factor (Mex67), an mRNA localization factor (Hek2), a cytoplasmic translation factor (Tif1), and cytoplasmic mRNA turnover and surveillance factors (Xrn1 and Ski2). Hrp1 and Nab2 function in mRNA cleavage and polyadenylation but additional roles are reported for Nab2 in mRNA packaging (Batisse et al., 2009), export (Iglesias et al., 2010), and nuclear surveillance (Schmid et al., 2012), and for Hrp1 in nuclear and cytoplasmic surveillance (González et al., 2000; Kuehner and Brow, 2008).

For target site identification, we used the crosslinking and analysis of cDNA (CRAC) technique (Granneman et al., 2011). Actively growing cells expressing HTP-tagged (His_6-TEV-Protein A) proteins under the control of the endogenous promoter were UV irradiated to fix direct protein:RNA contacts. After stringent, multi-step affinity purification, mild RNase digestion, and radiolabelling, RNPs were isolated by SDS-PAGE (Figure S1A available online). Bound RNA fragments were amplified by RT-PCR and analyzed by high-throughput sequencing. Identical conditions were used for all proteins tested, and in most cases replicate data sets acquired (Table S1). We also repeated the analysis for the poly(A)-binding protein Nab2 in an *rrp6Δ* background, which is reported to stabilize its transient binding (Schmid et al., 2012). Comparison of the number of reads mapping to each annotated transcript in replicate data sets revealed good reproducibility, with most Spearman rank correlation coefficients $\rho \geq 0.75$ (Figure S1B). Furthermore, the most highly enriched 10% of mRNAs in Hrp1, Nab2, and Hek2 data sets showed significant overlap with published immunoprecipitation analyses ($\chi^2 \leq 0.001$) (Batisse et al., 2009; Hasegawa et al., 2008; Kim Guisbert et al., 2005).

A breakdown of hits by transcript class revealed a broad range of substrate specificities for the tested proteins (Figure S1C). The poly(A)-binding protein Pab1 predominantly bound to mRNAs, whereas tRNAs were prevalent targets of the nuclear surveillance factor Mtr4, consistent with reports of extensive nuclear pre-tRNA degradation (Gudipati et al., 2012b). In Hrp1 data sets, snoRNAs were abundant, supporting a role in snoRNA biogenesis originally indicated by a ChIP study (Kim et al., 2006). Many proteins crosslinked to rRNAs at regions distinct from those typically detected as background (Figure S1D). Mex67 is reported to bind 60S and 40S (Faza et al., 2012) preribosomal particles, in good agreement with our data, and the peak of Xrn1 binding in ITS1 supports its role in cytoplasmic degradation of this excised spacer region. The nuclear surveillance factors Mtr4 and Trf4 assist in the degradation of the 5′ ETS, and we identified binding within this region. The specific rRNA association of the cytoplasmic helicase Ski2 is likely to

reflect interactions with translating ribosomes during mRNA degradation by the exosome. Other proteins showed distributed binding on the pre-rRNA (Figure S1D), but the significance is currently unclear.

Assembly and Architecture of mRNPs

Analyses of hits in mRNAs provided a high-resolution picture of the dynamic assembly of mRNPs. For each protein, we calculated the ratio of reads mapping across exon-exon (EE) versus intron-exon (IE) junctions (Figure 1B) (Schneider et al., 2012). IE junctions are exclusively present in unspliced pre-mRNA, and EE junctions in spliced mature mRNAs, so the EE/IE score indicates when, relative to splicing, each protein associates with the mRNP. Low scores were found for the nuclear cap-binding protein Cbc1, TREX components Tho2 and Gbp2, and nuclear surveillance factors Mtr4 and Trf4, consistent with these factors acting during or shortly after transcription. In contrast, high scores for the translation initiation helicase Tif1 and cytoplasmic surveillance factors Xrn1 and Ski2 indicate that they function late in the mRNP lifecycle. The slightly lower score for Xrn1 is consistent with its role in surveillance of unspliced pre-mRNAs and lariat intermediates (Hilleren and Parker, 2003).

The EE/IE analysis was most informative for shuttling proteins. For example, Mex67 and Nab2 load onto mRNA in the nucleus and contribute to export (Iglesias et al., 2010) and are removed at the cytosolic face of the nuclear pore (Lund and Guthrie, 2005; Tran et al., 2007). The lower EE/IE scores for Nab2 suggest that Nab2 enters the mRNP before Mex67, which is perhaps only recruited when cleavage and polyadenylation is complete. Nab2 and Pab1 are both implicated in poly(A) tail length control, but it has been unclear which acts first. The EE/IE score for Pab1 is lower than that of Mex67 and similar to that of Nab2, supporting early roles for both Nab2 and Pab1 in the nucleus. The cleavage factor Hrp1 also shuttles and can contribute to cytoplasmic surveillance (González et al., 2000). The low EE/IE score, however, suggests that Hrp1 primarily functions early in mRNP biogenesis, consistent with prolonged cytoplasmic binding of Hrp1 denoting an aberrant mRNP. Conversely, the high score for Hek2, which contributes to translational regulation and mRNA localization (Irie et al., 2002; Paquin and Chartrand, 2008), suggests that it binds late in the nucleus and is predominantly associated with cytoplasmic mRNPs.

Although the architecture of several ribonucleoprotein complexes, such as the ribosome and spliceosome, has been studied in detail, little is known about the topology of mRNPs. We therefore examined their organization by plotting the average binding distribution (hit density) of each protein across its top 1,000 mRNA targets (Figure 1C) and across individual mRNAs (Figures 1J–1L). Cbc1 and Tif1 reside in complexes that interact with the 5′ cap and predominantly crosslinked to mRNA 5′ ends, whereas the poly(A)-binding protein Pab1 bound at mRNA 3′ ends. For the TREX components Gbp2 and Tho2 and export receptor Mex67 hits mapped across mRNA bodies, consistent with their proposed ability to bind at multiple sites. The even hit distribution for Mex67 suggests that it binds full-length mRNAs, whereas the 5′ enrichment for Gbp2 and Tho2 presumably reflects binding to nascent transcripts at various stages of elongation. The mRNA localization factor Hek2 is homologous to the human poly(C)-binding proteins and preferentially binds $(CNN)_n$ motifs (Hasegawa et al., 2008; Wolf et al., 2010). Consistently, Hek2 showed no overall positional bias but a strong specificity for CNN repeats within individual mRNAs (Figure 1L).

Nab2 and Hrp1 contribute to mRNA cleavage and polyadenylation and, consistent with this, bound the 3′ ends of mRNAs. Unexpectedly, they also recovered many 5′-proximal RNA fragments, as did the surveillance factors Trf4 and Mtr4 (Figure 1C) and the exosome-associated nucleases Rrp44 and Rrp6 (Schneider et al., 2012), which participate in 3′-5′ nuclear decay. We propose that this reflects binding to unstable promoter-proximal RNA fragments rather than to the 5′ ends of full-length mRNAs (see below). Nab2 binding was observed throughout the body of mRNAs, consistent with previous ChIP analyses (González-Aguilera et al., 2011). In addition to binding poly(A), Nab2 shows nonspecific RNA-binding activity (Viphakone et al., 2008) and may be an architectural component of mRNPs (Batisse et al., 2009).

In the cytoplasm, the 5′ to 3′ and 3′ to 5′ mRNA decay pathways are preceded by deadenylation of the poly(A) tail to ~10–12 nt. Pab1 is then displaced and the 3′ end becomes accessible to the Ski2/3/8 complex and exosome for 3′ degradation, or the Lsm1-7/Pat1 complex that activates decapping and 5′ degradation by Xrn1. We observed prominent peaks at the 3′ end of mRNAs for Ski2 and, less expectedly, for the 5′ to 3′ exonuclease Xrn1. This indicates that the oligo(A) tail is the site of a rate-limiting step in mRNA turnover, perhaps reflecting assembly of the surveillance machinery or regulated initiation of decay. Indeed, Xrn1 interacts with the Lsm1-7/Pat1 complex that crosslinks primarily to mRNA 3′ ends (Mitchell et al., 2013). The 3′ peak of Xrn1 hits might also reflect slowed degradation of the 3′UTR due to the presence of RNA-binding proteins not displaced by translating ribosomes. The absence of clear peaks of Xrn1 crosslinking elsewhere along the mRNA body is consistent with its high processivity, perhaps following the last translating ribosome (Hu et al., 2009). Supporting this model, we observed a moderate accumulation of Xrn1 upstream of mRNA stop codons (Figure S2A). Conversely, Ski2 binding was distributed across the body of mRNAs, suggesting that 3′-5′ decay is slower or is more prone to pausing, perhaps due to collisions with translating ribosomes.

RNP Composition Defines Distinct Transcript Classes

Having obtained a picture of an "average" mRNP, we next investigated how RNP composition varies between mRNAs and between classes of Pol II transcripts. For each protein tested, we extracted all hits in Pol II transcribed RNAs (Table S1) and plotted the proportion mapping to snoRNAs, CUTs, SUTs, XUTs, and two mRNAs. Combining the data for all 13 proteins produced "RNP profiles" for these six transcript types (Figures 1D–1I). Cbc1, Gbp2, and Tho2 were moderately abundant in all six RNP profiles and bind with similar distributions along mRNAs, CUTs, and SUTs (Figures 1M and S2B), suggesting they are universal RNP components and that early RNP assembly is similar for mRNAs and lncRNAs. These data corroborate reports that the CBC is present in snoRNP assembly intermediates (Schwer et al., 2011), and that the THO complex regulates snoRNA expression by binding at the 3′ end (Figure S2C) (Larochelle

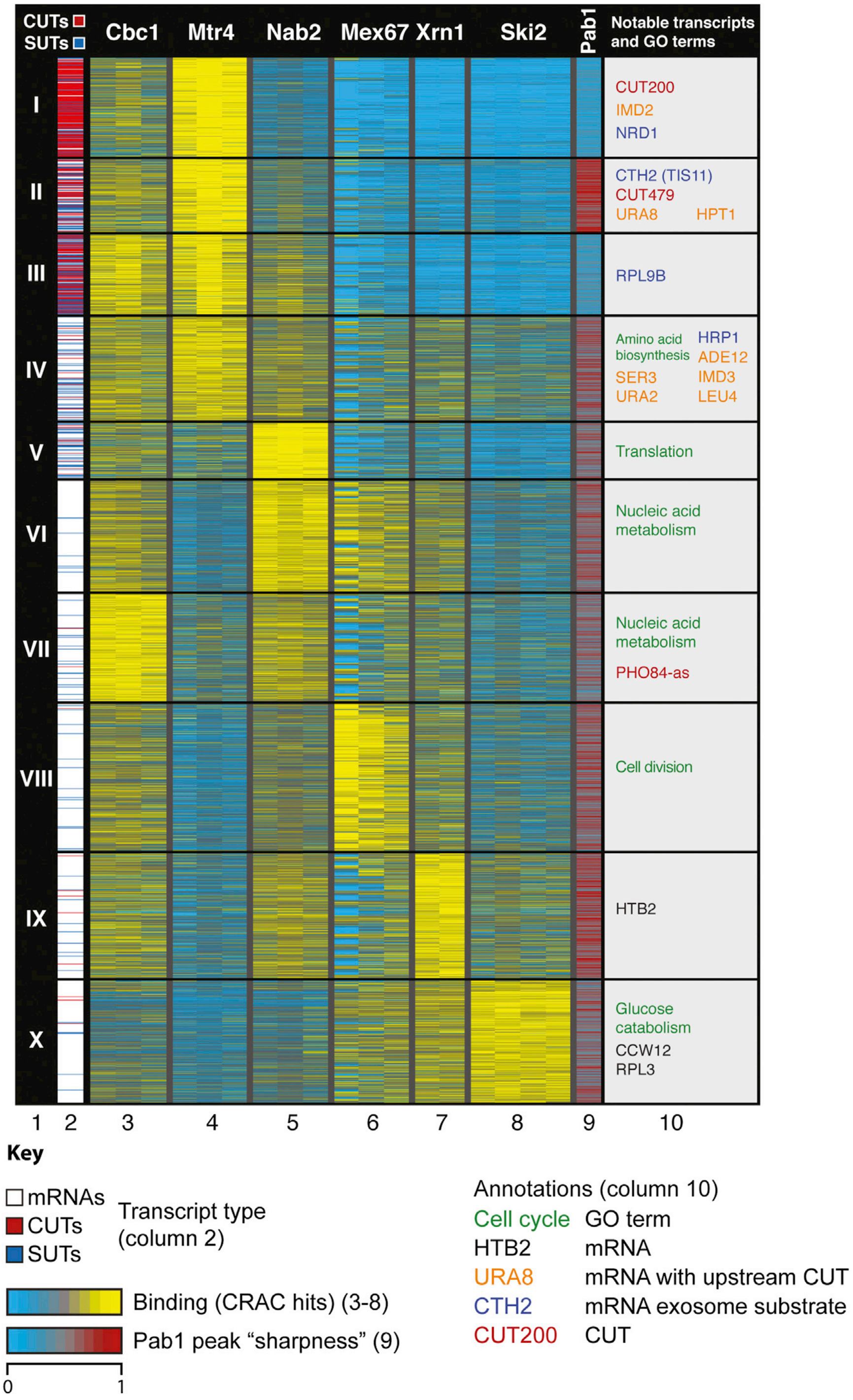

Figure 2. RNP Composition Reflects Transcript Function and Reveals Distinct RNA Classes

Transcripts arranged by k-medians clustering (n = 4960; k = 10, column 1) based upon their binding to maturation and turnover factors. Column 2: location of CUTs (red), SUTs (blue) and mRNAs (white). Columns 3–8: relative number of hits (per million hits in Pol II transcribed RNAs) in Cbc1, Mtr4, Nab2, Mex67, Xrn1, and Ski2 data sets for each transcript. Column 9: Pab1 peak score, reflecting the specificity of Pab1 interaction. Column 10: significantly overrepresented GO terms (green) and individual transcripts referred to in the text (color coding is indicated in the key). See also Figure S2 and Tables S2, S3, S4.

et al., 2012). Hrp1 and Nab2 have multiple functions in RNA metabolism and were present in snoRNPs, lncRNPs, and the *HTB2* mRNP, indicating that they too are ubiquitous constituents of RNPs.

Despite these similarities in early RNP assembly, there were striking differences in binding of different transcript classes to cytoplasmic and nuclear surveillance factors. *RPL3* was strongly bound by Xrn1 and Ski2, but not Mtr4 or Trf4, indicating that it is predominantly degraded in the cytoplasm, whereas the reverse was seen for snoRNAs, SUTs, and particularly CUTs. Furthermore, *RPL3* and *HTB2* were bound more extensively by the export receptor Mex67 than were snoRNAs, CUTs, and SUTs. Collectively, these data suggest that CUTs and SUTs, like snoRNAs, are predominantly confined to the nucleus and that the distinction between lncRNPs and mRNPs occurs after early RNP packaging but prior to Mex67 recruitment. Notably, CUTs and SUTs were significantly bound by Pab1, Hrp1, and Nab2 suggesting that 3′ end formation on lncRNAs initially resembles that of mRNAs but culminates in nuclear retention rather than export.

In addition to differences in RNP composition between transcript classes, we identified heterogeneity within each class. For example, Trf4 bound to *HTB2* more strongly than *RPL3*, consistent with reports that Trf4 regulates *HTB2* expression (Reis and Campbell, 2007). This heterogeneity raised the question of whether the scarce lncRNA hits in Xrn1, Ski2, and Mex67 data sets arise from a general low level of binding to lncRNAs or robust interactions with a few atypical lncRNAs. To assess heterogeneity among mRNPs and lncRNPs, we performed a k-medians clustering analysis of mRNAs, CUTs, and SUTs based upon their individual RNP profiles (Figure 2A). These profiles were derived from the number of hits for each transcript in Cbc1, Mtr4, Nab2, Mex67, Xrn1, and Ski2 data sets (Figure 2A, columns 3–8), with hits normalized for each row (transcript). Within the Pab1 data set, some transcripts with relatively low numbers of total hits displayed a sharp peak of binding at the 3′ end. To distinguish

site-specific binding at these putative polyadenylation sites, from broadly distributed, potentially nonspecific, interactions across transcript bodies, we generated a “peak sharpness” score (Figure 2A, column 9). For this, we identified the highest peak in each transcript and divided the value of this by the maximum obtained when reads in the surrounding 400 nt region were randomly placed.

The cluster analysis recapitulated the major findings from Figure 1, with CUT and SUT classes of lncRNAs predominantly falling into clusters (I–III) distinct from mRNAs (IV–X). Furthermore, whereas cluster I was enriched for CUTs, clusters II and III contained more SUTs (Table S2), revealing differences not readily apparent from the class-wide analysis in Figure 1. In comparison to the “CUT cluster” (I), the “SUT clusters” (II and III) had more specific Pab1 binding. Cluster III also has less dominant binding to the nuclear surveillance factor Mtr4. This suggests that although all lncRNAs are predominantly retained and degraded in the nucleus, CUTs are more rapidly degraded so might represent byproducts of functional transcription, whereas SUTs are more stable so perhaps function as transcripts. Furthermore, 34.4% of SUTs fell into mRNA clusters (IV–X), compared to just 6% of CUTs, indicating that CUTs are distinct from mRNAs but SUTs and mRNAs often overlap.

The PHO84-as lncRNA, which falls into an mRNA cluster, functions in *trans* (Camblong et al., 2009) and accumulates in the cytoplasm (Castelnuovo et al., 2013). Furthermore, “mRNA-like” SUTs (clusters IV–X) bound more strongly to Mex67 than SUTs in the lncRNA clusters (I–III) (Figure S2D). The “mRNA-like” lncRNAs therefore appear to behave and function differently from the “standard” lncRNAs in clusters I–III, perhaps representing functional transcripts exported to the cytoplasm. Indeed, inspection of published transcriptome profiling data revealed that cluster IV–X SUTs accumulate less than cluster I–III SUTs in nuclear surveillance mutants (Figure S2E) (Gudipati et al., 2012b), suggesting they are degraded in the cytoplasm by Xrn1, with which they interact (Figure 2A, column 7). Hek2 has a role in mRNA stabilization (Mauchi et al., 2010; Vogel et al., 2011) and localization, and SUTs in clusters IV–X bound more abundantly to Hek2 than those in clusters I–III (Figure S2D). Hek2 might therefore be one factor that helps discriminate mRNAs and “mRNA-like” lncRNAs from “standard” lncRNAs, based on sequence-specific binding. Indeed, SUTs in clusters VI–X have particularly long CNN repeats (Figure S2F) to which Hek2 binds (Figure S2G).

Clusters I–III contained 411 mRNAs (Table S2), and these are likely to behave like lncRNAs, with retention and/or degradation in the nucleus. Rrp6 is active in CUT surveillance (Neil et al., 2009; Xu et al., 2009), and cluster I–III mRNAs are highly stabilized in strains lacking this exonuclease (Figure S2D). Furthermore, *NRD1* (cluster I) transcripts undergo attenuation dependent on the Nrd1–Nab3 complex (Kuehner and Brow, 2008), which functions in the termination and nuclear surveillance of many CUTs. Additional cluster I–III mRNAs, such as *CTH2* (Ciais et al., 2008) and *RPL9B* (Gudipati et al., 2012a) are also terminated and processed/degraded via Nrd1-dependent pathways. Other cluster I–III mRNAs including *URA8* and *IMD2* are regulated by promoter-proximal CUTs (Kuehner and Brow, 2008; Thiebaut et al., 2008). We predict that the remaining cluster I–III mRNAs either behave like lncRNAs, with retention and degradation in the nucleus, or are regulated by overlapping lncRNAs. Indeed, comparison to ribosome profiling data (Brar et al., 2012) (Figure S2H) reveals that cluster I–III mRNAs are ~5-fold less abundant on ribosomes than cluster IV–X mRNAs, relative to their transcription rate (gauged by Gbp2 binding; Figure S2H).

We noted significant heterogeneity between the mRNA clusters (IV–X). Cluster IV was most similar to the lncRNA clusters (I–III), with binding to Mtr4 as well as cytoplasmic surveillance factors. Several cluster IV mRNAs overlap CUTs (*URA2*, *SER3*, *ADE12*, *IMD3*, and *LEU4*) (Davis and Ares, 2006; Thiebaut et al., 2008), and we suggest that the “mixed” RNP profile of cluster IV reflects genes where mRNAs and lncRNAs are transcribed concurrently (perhaps in distinct subpopulations of cells). GO term analyses (Table S3) revealed that some mRNA clusters were enriched for transcripts related to particular cellular processes, suggesting that mRNP composition is linked to the function of the encoded protein. For example, transcripts encoding ribosomal proteins were prevalent in cluster V, which showed high binding by Nab2 (Figure 2A) and strong stabilization in Rrp44 mutants (Figure S2D) (Gudipati et al., 2012b). This is consistent with reports that Nab2 and Rrp44 act in the nuclear surveillance of ribosomal protein gene pre-mRNAs (Bousquet-Antonelli et al., 2000; Gudipati et al., 2012b; Schmid et al., 2012). Transcripts encoding proteins with functions critical to the nucleus, such as nucleic acid metabolism, were enriched in clusters VI and VII, with high Cbc1 and Nab2 binding, whereas those encoding proteins participating in predominantly cytoplasmic processes such as glucose metabolism were enriched in cluster X, with high levels of Ski2, Xrn1, and Mex67 binding. This suggests that mRNAs are preferentially regulated in the cellular compartment most appropriate to the function of their encoded protein, perhaps facilitating rapid feedback regulation.

RNA Classes Are Defined by Distinct Modes of 3′ End Formation

The largely distinct behavior and RNP composition of mRNAs, CUTs, and SUTs lead us to question how they are distinguished in the cell. Both CUTs and SUTs bound early, cotranscriptionally recruited, mRNP packaging components (Cbc1, TREX, Hrp1, and Nab2) but were underrepresented (particularly CUTs) among Mex67 targets. These results indicated that the distinction between CUTs, SUTs, and mRNAs is made following transcription elongation but prior to the acquisition of export competence. This suggested that there might be crucial differences in 3′ end formation, which generally proceeds via one of two possible mechanisms: stable mRNA 3′ ends are generated via cotranscriptional cleavage and polyadenylation, whereas the 3′ ends of some CUTs arise directly from Nrd1-dependent transcription termination coupled to oligoadenylation and turnover. Formation of the 3′ ends of SUTs has not been studied in detail.

Plotting the average distribution of Pab1 hits across mRNAs, CUTs, and SUTs (Figure 3A, red) revealed 3′ peaks for mRNAs and SUTs but distributed binding across CUTs, consistent with the lower Pab1 peak scores in the CUT cluster (Figure 2, cluster I). Thus 3′ end processing only of mRNAs and SUTs results in the acquisition of a stable, Pab1-bound poly(A) tail.

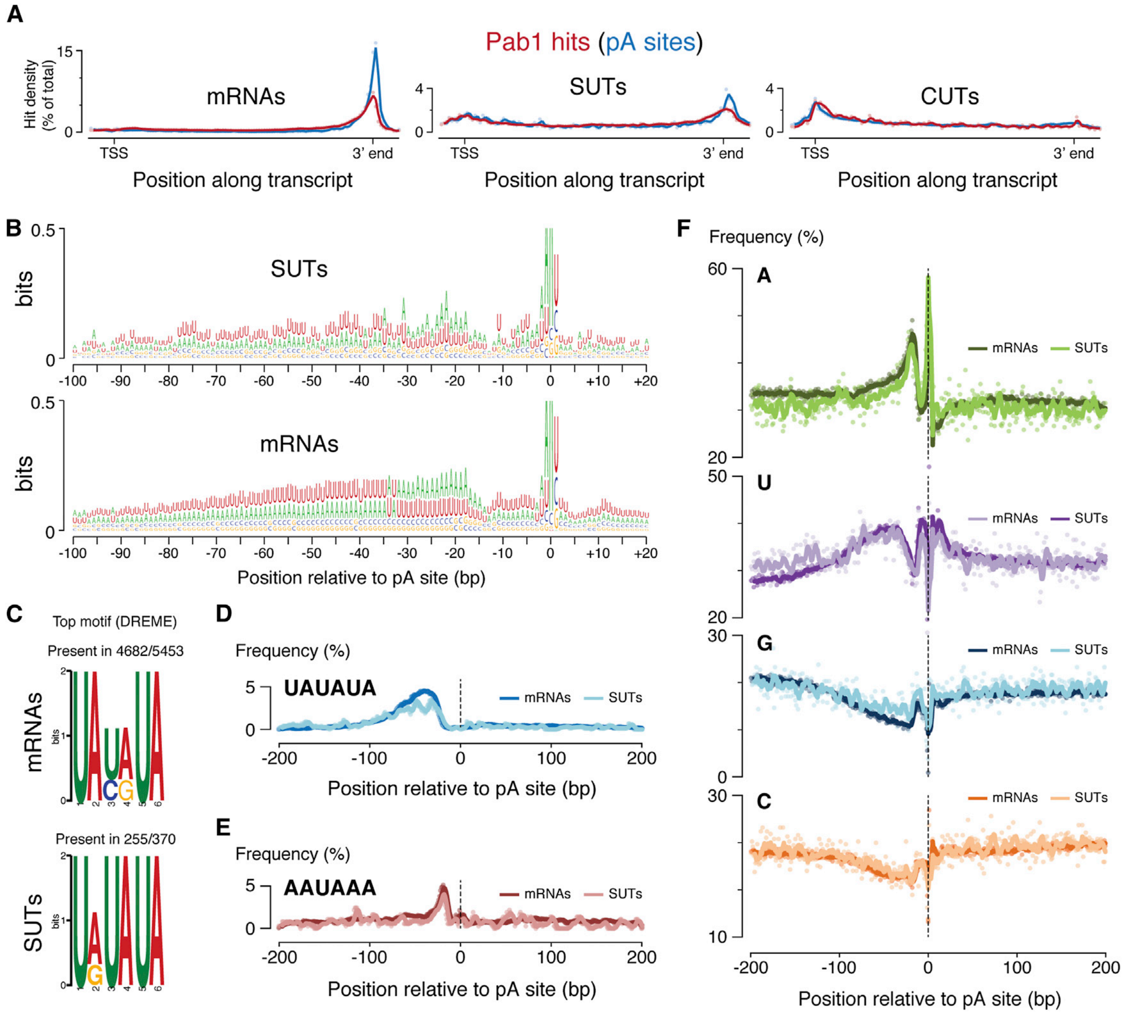

Figure 3. RNA Classes Are Defined by Alternative Modes of 3′ End Formation

(A) Red: average distribution of Pab1 hits across mRNAs, SUTs, and CUTs (300 nt flanks included). Blue: average distribution of poly(A) (pA) sites, defined as the last genome-encoded nucleotide within Pab1-bound RNA fragments with a non-genome-encoded poly(A) tail.

(B–F) Analysis of the set of genomic sequences flanking the most frequent pA site for each mRNA (n = 5,453) or SUT (n = 370). (B) Sequence logo of these regions. (C) Sequence logo of the most highly enriched motif in these regions. (D and E) Occurrence of UAUAUA and AAUAAA motifs. (F) Nucleotide frequency distributions.

Together with the clustering analysis in which SUTs and mRNAs overlapped and were distinct from CUTs, this leads to a working model in which SUTs undergo cleavage and polyadenylation like mRNAs. In contrast, CUTs are terminated by a distinct mechanism, most likely dependent on Nrd1-Nab3, coupled to rapid turnover.

Cleavage and polyadenylation of pre-mRNAs is dependent on the recognition of a precise configuration of sequence motifs by a large multicomponent complex. To investigate whether SUT 3′ ends are defined by similar motifs, we needed to precisely localize sites of 3′ end formation on SUTs and mRNAs. We therefore searched the Pab1 data set for chimeric reads in which the mapped sequence is followed by adenosine residues that are not genome encoded (nonencoded A-tails). These represent junctions between mRNA 3′ ends and poly(A) tails, and extracting the genomic coordinate of the last encoded nucleotide provided us with a transcriptome-wide set of precise poly(A) (pA) sites. Plotting these sites across mRNAs and SUTs gave sharp 3′ peaks (Figure 3A, blue), indicating that 3′ ends were detected with high precision. For many transcripts, we found multiple pA sites, consistent with a recent transcript isoform sequencing study (Pelechano et al., 2013) and indicating that alternative pA sites are prevalent. To compare the sequence features defining mRNA and SUT 3′ ends, we generated logos for the genomic sequence flanking the most frequently identified pA site for each mRNA and SUT (Figure 3B). These were similar for mRNAs and SUTs, with an AU-rich region extending ~80 nt upstream of the pA site, and a bias toward adenosine as the last encoded residue (or immediately 3′ to the last encoded residue, because these cannot be distinguished). A motif search identified

UAUAUA as highly enriched in the 3′ regions of SUT and mRNA genes, most frequently located ~30–70 nt upstream of the pA site (Figures 3C and 3D). This motif corresponds to the efficiency element, originally identified ~50 nt upstream of mRNA cleavage sites. Messenger RNA 3′ ends are also defined by the positioning element, AAUAAA, 10–30 nt upstream of the pA site, and U-rich regions flanking the pA site. We detected both of these features in genes encoding SUTs and mRNAs when we plotted the frequency of AAUAAA motifs (Figure 3E) or nucleotide base composition (Figure 3F) around pA sites. We conclude that mRNAs and SUTs possess stable Pab1-bound poly(A) tails, and their 3′ ends are defined by common sequence elements.

mRNA Cleavage and Polyadenylation Factors Participate in CUT Surveillance

The mode of 3′ end formation of mRNAs and SUTs is apparently distinct from CUTs, so it was surprising that CUTs bound to the mRNA cleavage and polyadenylation factors Hrp1 and Nab2. Hrp1 specifically binds the UAUAUA efficiency element via its tandem RRM domains (Pérez-Cañadillas, 2006). This motif was enriched at Hrp1-binding sites in mRNAs but also within SUTs and CUTs (Figure 4A). Single-nucleotide deletions in cDNA reads indicate the precise nucleotide crosslinked to the bait protein, and for Hrp1 hit density and sequence deletions were elevated over UAUAUA motifs in all three classes of transcript indicating that this is a direct binding site (Figure 4B). However, whereas mRNAs and SUTs displayed a 3′ peak of Hrp1 binding, CUTs did not (Figure 4C). Furthermore, ~40% of Hrp1-binding sites at the 3′ end of mRNAs and SUTs possessed a UAUAUA element, but there was no such enrichment among the low number of Hrp1-binding sites at the 3′ end of CUTs (Figure 4D). This indicates that Hrp1 binds directly to the efficiency element to promote 3′ processing of mRNAs and SUTs, but binds in a more distributed manner throughout CUTs. Notably, even within SUTs and mRNAs, the majority of Hrp1 is bound to promoter-proximal regions (Figure 4C), and ~90% of binding sites lack a UAUAUA motif (Figure 4A). This suggested that Hrp1 has additional functions unrelated to cleavage and polyadenylation and independent of binding to UAUAUA.

The poly(A)-binding protein Nab2 also participates in mRNA 3′ end formation and showed increased binding at the 3′ ends of mRNAs and SUTs. However, like Hrp1, Nab2 binding was more distributed over CUTs, with additional binding toward the 5′ ends of mRNAs and SUTs (Figure 4C). The lack of binding to the 3′ end of CUTs was not simply due to their rapid turnover, as a 3′ peak of Nab2 binding was still absent from full-length CUTs stabilized in an *rrp6Δ* strain (Figure 4C). We conclude that both Hrp1 and Nab2 function in mRNA and SUT 3′ end formation but act in a different manner on CUTs and promoter-proximal regions.

To compare the functions of Nab2 and Hrp1 when bound to mRNAs and CUTs, we constructed strains with endogenous *NAB2* and *HRP1* genes under the control of glucose-repressible P_{GAL} promoters. The effects of Hrp1 and Nab2 depletion were assessed for transcripts identified as targets in the CRAC analyses. Northern analysis of *CCW12*, which falls into a typical mRNA cluster (X), revealed a slight increase following Nab2 depletion and dramatic decrease upon Hrp1 depletion (Figure 4E). This is consistent with the essential role of Hrp1 in mRNA synthesis and with the mild general increase in mRNA expression observed in a previous analysis of Nab2 depletion (Schmid et al., 2012). In contrast, the abundance of two tested CUTs, CUT479 and CUT200, was increased up to 15-fold following depletion of Nab2 or Hrp1 (Figure 4E). Many of the RNA fragments isolated with Hrp1 or Nab2, including those mapping to CUTs, possessed short nonencoded oligo(A) tails (Figures 5A–5C). These are hallmarks of nuclear decay intermediates, suggesting that CUTs bound by Hrp1 and Nab2 were undergoing active degradation.

Previous analyses showed that Nab2 binds the surveillance factors Rrp6 and Trf4 (Schmid et al., 2012) and participates in degradation of intron-containing pre-mRNAs, whereas Hrp1 was implicated in Nrd1-dependent termination coupled to pre-mRNA turnover at the *NRD1* and *HRP1* loci (Kuehner and Brow, 2008). We conclude that Hrp1 and Nab2 participate in the nuclear turnover of CUTs, in addition to their roles in the generation of stable mRNAs and perhaps SUTs. These dual roles support a model in which 3′ end processing is a key step in determining transcript fate.

Early Termination Generates Promoter-Proximal lncRNAs

Finally, we sought to determine the origin of the 5′ proximal binding of surveillance factors to mRNAs. RNAs carrying nonencoded A-tails were identified for many proteins (Figure 5A) and indicate that transcripts have been released from the polymerase. Generally, stable mRNAs possess long Pab1-bound poly(A) tails that promote export and translation but are deadenylated to ~10–12 adenosines prior to cytoplasmic turnover by Xrn1 or the Ski complex and exosome. In contrast, short (4–5 nt) oligo(A) tails mapping throughout a gene arise from the adenylation activity of the TRAMP complex and characterize nuclear surveillance intermediates. In agreement with these roles, (1) A-tails in Xrn1 and Ski2 data sets were ~1–12 nt long (Figures 5B and 5C), absent from transcript classes such as tRNAs (Figure 5D), and almost exclusively present at the 3′ end of mRNAs (Figure 5E); (2) Pab1 bound to long poly(A) tails (present on 74.2% of recovered fragments) but not oligo(A)$_{<10}$ (Figures 5B and 5C); and (3) Mtr4 substrates universally possessed short (4–5 nt) oligo(A) tails (Figures 5B–5D), some of which mapped across mRNAs (Figure 5E). Therefore, oligo(A) tails are a universal feature of TRAMP activity and are exclusively associated with nuclear surveillance, whereas the longer A-tails in Xrn1 and Ski2 data sets reflect 3′ poly(A) tails on mRNAs and some other Pol II transcripts. The abundant short oligo(A)-tails in Nab2 data sets (Figure 5B) support a noncanonical role in surveillance.

Most oligo(A)-tailed mRNA reads in Mtr4 data sets mapped to promoter-proximal regions (Figure 5E) and even for long genes did not extend beyond the first ~400 nt (Figure 5F). Promoter-proximal Mtr4-bound fragments are therefore unlikely to represent decay intermediates of full-length mRNAs but might instead arise from high levels of early transcription termination. In Nab2 data sets, A-tails were shorter for promoter-proximal reads than for reads mapping to mRNA 3′ ends and largely absent from reads mapping to central regions of mRNAs (Figure 5G). Nab2 therefore binds to poly(A) tails on full-length mRNAs, and

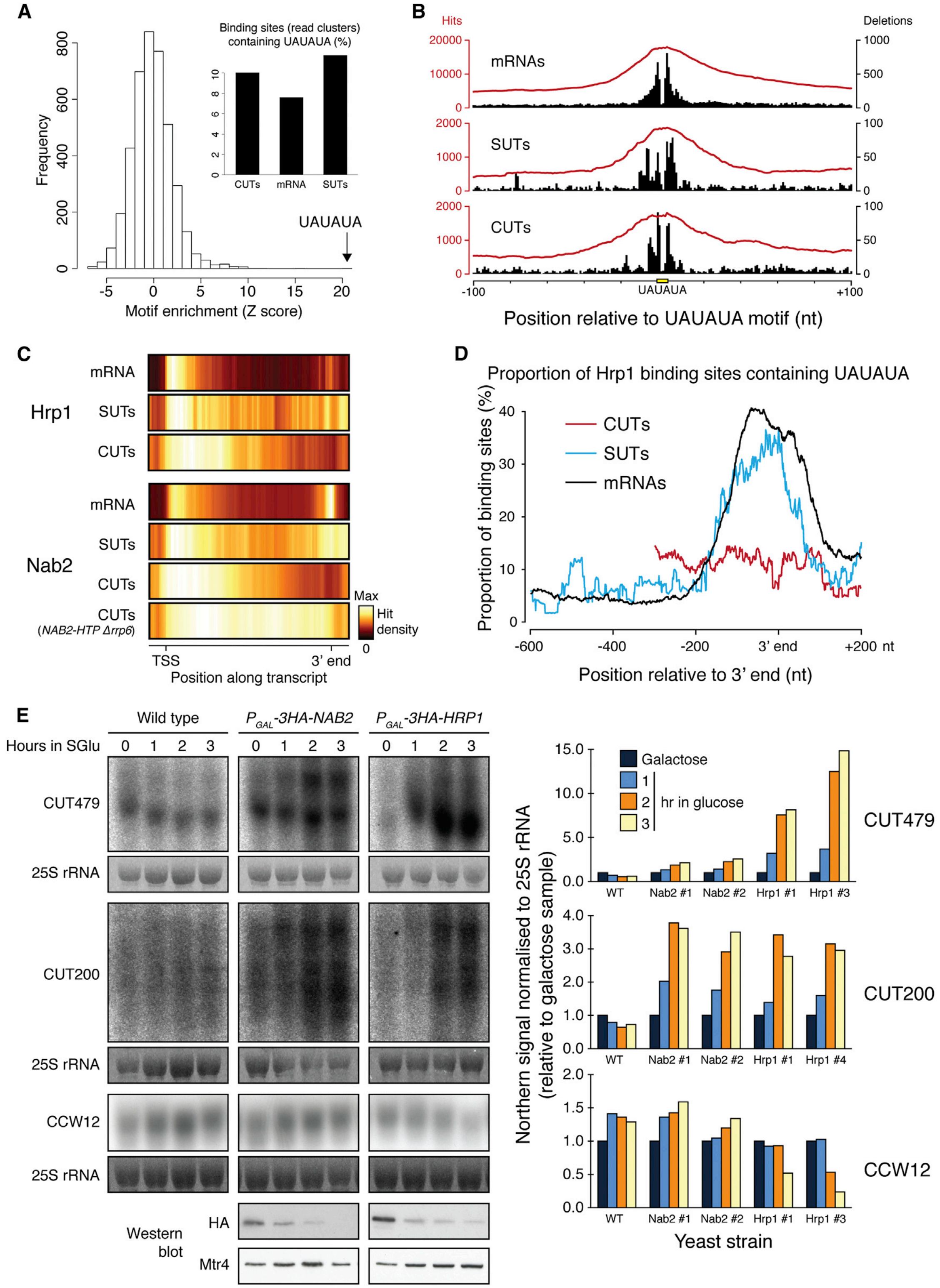

Figure 4. Dual Roles of Hrp1 and Nab2 in mRNA Cleavage/Polyadenylation and in CUT Surveillance

(A) Enrichment scores for 6 nt motifs in Hrp1-bound RNA fragments. Inset: proportion of Hrp1-binding sites containing the UAUAUA motif in mRNAs, CUTs and SUTs.

(B) Distribution of Hrp1 hits (black) and deletions (red) around UAUAUA motifs in mRNAs, CUTs, and SUTs.

(legend continued on next page)

to oligo(A)-tailed unstable transcripts from promoter-proximal regions, whereas interactions with mRNA central regions apparently reflect an adenosine-independent-binding activity. Promoter-proximal transcripts of a similar length were previously identified in yeast depleted of Rrp6 and Trf4, which stabilizes full-length CUTs (Figure 5H) (Neil et al., 2009). This supports the notion that promoter-proximal transcripts do not originate from longer precursors.

To test whether promoter-proximal fragments arise from early termination, we compared the distribution of Pol II (Churchman and Weissman, 2011), the termination factors Nrd1 and Nab3 (Figure 5H) (Wlotzka et al., 2011), and the 5′ exonuclease Rat1 (Granneman et al., 2011) across mRNAs. The promoter-proximal enrichment of Pol II has been interpreted as stalled elongation complexes that are competent to resume transcription. However, Nrd1, Nab3, and Rat1 are all enriched in this region, which coincides with the promoter-proximal oligoadenylated fragments. This suggests that stalled Pol II is susceptible to early termination, triggered either by Nrd1-Nab3 or by cleavage/decapping followed by a Rat1-dependent "torpedo" mechanism. The resultant 5′ fragments account for most Mtr4, Trf4, Nab2, and Hrp1 hits mapping to protein-coding genes, thus removal of early termination products is a major function of the nuclear surveillance machinery. Furthermore, these transcripts bind the same factors as CUTs (Mtr4, Trf4, Hrp1, Nab2), indicating that protein-coding loci give rise to both classical mRNPs and an abundant class of promoter-proximal transcripts that assemble and behave like unstable lncRNAs.

DISCUSSION

Our data reveal that distinct transcript classes are defined during 3′ end formation, with RNP compositions tailored to the functions and fates of the transcripts. These classes loosely align with existing annotations, but we identified hundreds of exceptions. To address the extensive overlap between, and heterogeneity within, annotated transcript classes, we suggest an improved RNP-based classification, which reflects how transcripts are regulated and how they might function.

Tailored RNP Composition

There has been much debate about the function of pervasive transcription in eukaryotes. We find that CUTs and SUTs are predominantly retained and degraded in the nucleus, suggesting that their functions primarily arise from the act of transcription rather than the transcript itself. This refutes the notion that SUTs are generally "stable," but agrees with recent analyses identifying SUTs among exosome substrates (Gudipati et al., 2012b; Schneider et al., 2012). However, SUTs were mildly less prone to nuclear turnover than were CUTs, and their RNP composition overlapped with that of mRNAs, suggesting that some SUTs might function as stable transcripts. Xrn1 and the cytoplasmic exosome do not appear to function widely in bulk lncRNA turnover, but we suggest they degrade mRNA-like SUTs and provide a fail-safe for leaky nuclear surveillance. A substantial lncRNA class, termed XUTs, was reported to be exported and degraded in the cytoplasm by Xrn1 (van Dijk et al., 2011), but we see little evidence for this (Figure 1I). We also uncovered extensive heterogeneity in mRNP composition, with one mRNA class regulated by lncRNAs or subject to lncRNA-like turnover in the nucleus, and another regulated by Nab2 and Rrp44 (Gudipati et al., 2012b; Schmid et al., 2012). Other mRNAs were primarily subject to cytoplasmic regulation. We suggest that tailored RNP compositions enable transcripts to be regulated and localized in a way appropriate to their function (or that of the encoded protein).

The Multicolored Transcriptome

Conventional transcriptome profiling experiments (e.g., RNA-Seq) struggle to distinguish overlapping transcripts, particularly where one is less abundant. By combining high-resolution binding data for many RNP proteins, we obtained a "multicolored" view of the transcriptome and could readily distinguish overlapping transcripts with different RNP profiles. Most strikingly, this revealed that the nuclear surveillance machinery targets a major class of promoter-proximal lncRNAs apparently generated by early transcription termination and with an RNP composition resembling CUTs.

Our analyses suggest that this early termination is prevalent for "lncRNA-like" mRNAs, but occurs to some extent for most mRNAs, and we speculate that this reflects a checkpoint in Pol II transcription. Mtr4 hits peaked within ~150 nt of mRNA TSSs, coincident with locations of Pol II pausing (Churchman and Weissman, 2011). Here, transcription initiation factors exchange for elongation factors (Mayer et al., 2010) prior to the polymerase traversing the +2 nucleosome dyad ~90 nt further downstream, which can impede elongation. If remodeling of the transcription complex is unsuccessful or slow, we suggest that Pol II transcription is terminated. Termination might involve Nrd1, Nab3, and/or Rat1, which crosslink to promoter-proximal regions (Creamer et al., 2011; Wlotzka et al., 2011 and unpublished data) and are implicated in the early termination of some mRNAs and lncRNAs (Geisler et al., 2012). The oligoadenylated 3′ ends that we detect suggest that termination generates an entry site for the TRAMP and exosome complexes. This is consistent with either an Nrd1-dependent mechanism or endonuclease cleavage followed by Rat1-dependent termination, which was recently identified in humans (Wagschal et al., 2012). The distribution of Mtr4 hits suggests that this checkpoint is restricted to the first ~500 nt, consistent with the exclusion of termination factors from the midregions of genes by Y1P modification of the Pol II CTD (Mayer et al., 2012). The extent to which these early terminating transcripts function as ncRNAs remains to be determined.

(C) Average distribution of binding sites for Hrp1 (top) and Nab2 (bottom) across mRNAs, SUTs, and CUTs in the wild-type background. For Nab2 binding to CUTs an *rrp6Δ* strain is also shown.

(D) Frequency of UAUAUA motifs in Hrp1-binding sites near the 3′ end of mRNAs, CUTs, and SUTs (Xu et al., 2009).

(E) Northern analysis of CUT479, CUT200, and CCW12 abundance in wild-type, P_{GAL}-*NAB2* and P_{GAL}-*HRP1* strains after glucose-dependent repression. Northern signals were quantified for replicate experiments (right). Bottom: western analysis of HA-Hrp1, HA-Nab2, and Mtr4 abundance.

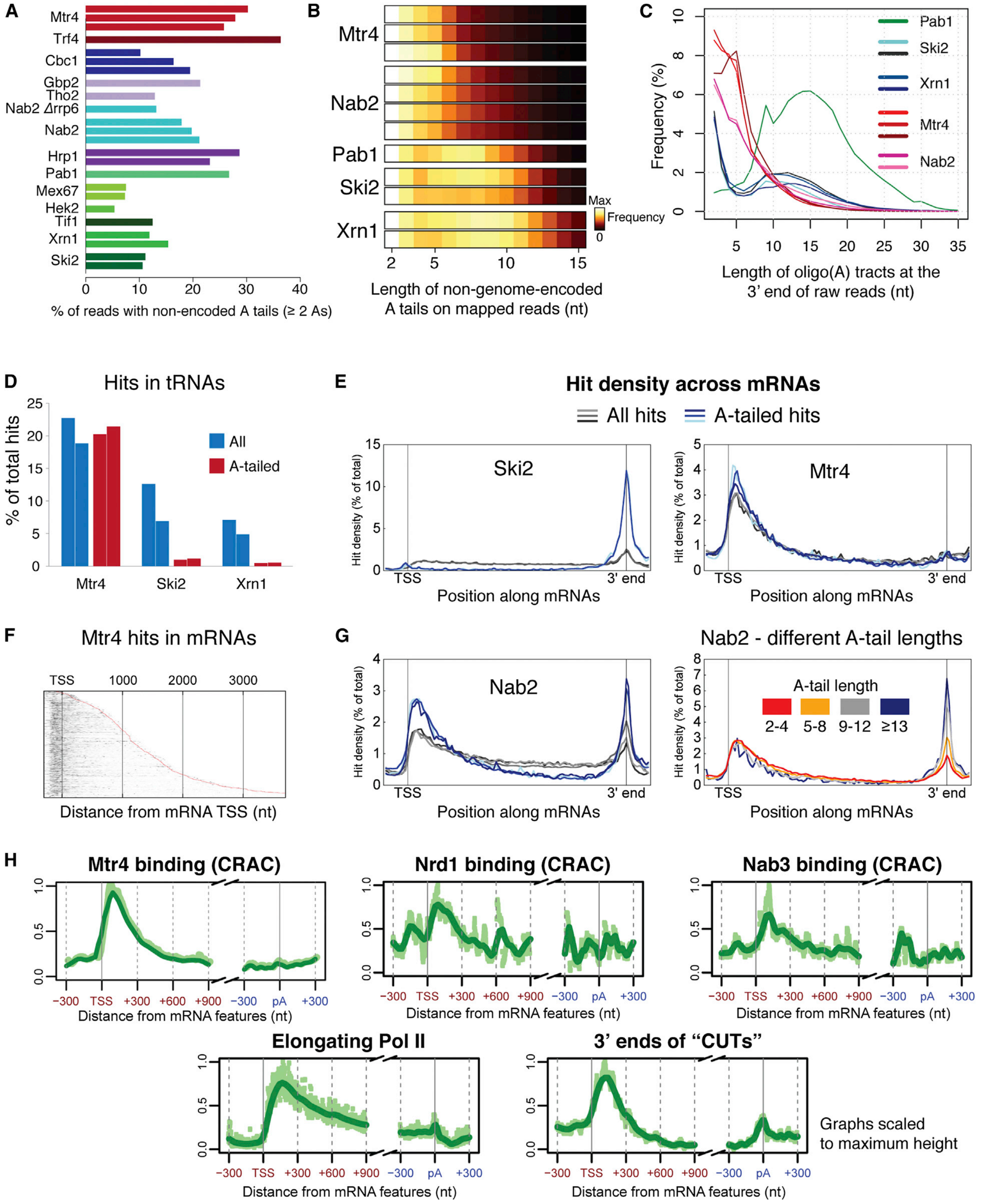

Figure 5. Nuclear Surveillance Factors Bind to Promoter-Proximal lncRNAs Arising from Early Termination within Protein-Coding Genes

(A) Frequency of non-genome-encoded A-tails in CRAC data sets.

(B) Length distribution of non-genome-encoded A-tails at the 3′ end of mapped reads in selected data sets (relative to the abundance of 2 nt tails).

(C) Length distribution of A-tails on raw reads (% of all analyzed reads). In comparison to (B), this analysis can detect longer A-tails but does not distinguish between genome-encoded and nonencoded tails.

(legend continued on next page)

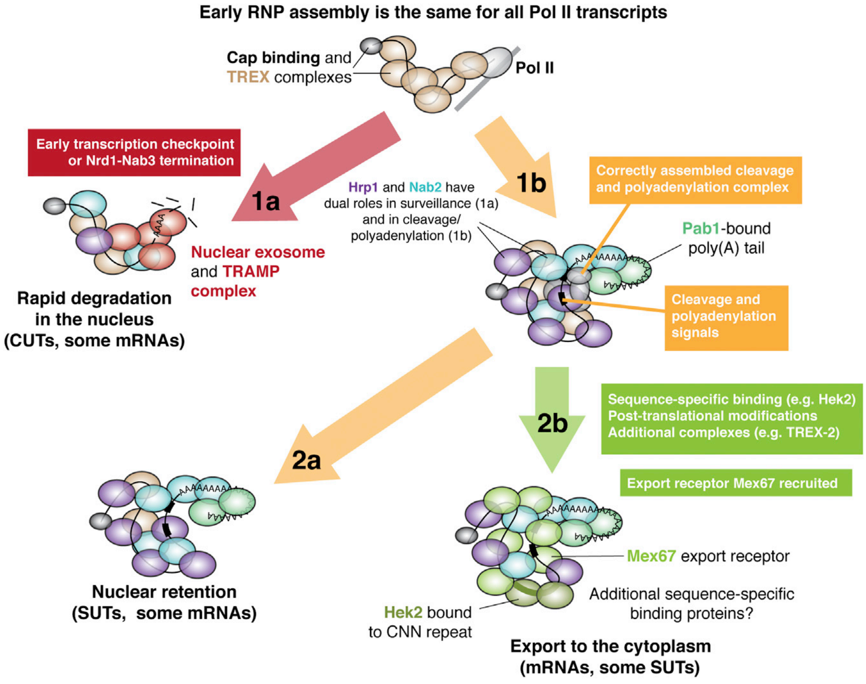

Figure 6. Determining the Fate of Pol II Transcripts

Early stages in RNP assembly (CBC and TREX binding) are the same for all Pol II transcripts. If transcription is terminated by Nrd1-Nab3 or at a putative promoter-proximal checkpoint (1a), the transcript is rapidly eliminated by the nuclear surveillance machinery. However, SUTs and mRNAs contain signals upon which the cleavage and polyadenylation machinery assembles (1b), and so acquire a stable Pab1-bound polyA tail. Most SUTs remain in the nucleus (2a), but mRNAs and some SUTs undergo additional events (e.g., sequence-specific binding by Hek2) that promote recruitment of the export receptor Mex67 (2b) and export to the cytoplasm. Approximately 10% of mRNAs behave like lncRNAs, with retention and degradation in the nucleus.

Determining Transcript Fate

Our analyses also revealed characteristics by which transcript classes are distinguished in the cell, with 3′ end formation emerging as a key step (Figure 6). For mRNAs and SUTs we detected the hallmarks of cleavage and polyadenylation, including an appropriate configuration of sequence elements, a Pab1-bound poly(A) tail, and Hrp1 bound to an efficiency element ~50 nt upstream of the pA site. In contrast, these were absent from CUTs, which therefore undergo a distinct termination pathway. This is most likely Nrd1-dependent termination, which is associated with exosome recruitment potentially explaining the inherently low stability of CUTs (Vasiljeva and Buratowski, 2006). The RNP composition of CUTs was related to that of snoRNAs (Figure 1), for which Nrd1-dependent termination is well established.

Analyses of two mRNA cleavage and polyadenylation factors, Hrp1 and Nab2, revealed additional roles in the surveillance of CUTs and promoter-proximal RNAs, supporting our conclusion that 3′ end formation is a key step in determining transcript fate. We propose that Hrp1 and Nab2 have dual-functions. Sequence nonspecific binding may be a default activity that is associated with recruitment of the nuclear surveillance system. In contrast, Hrp1 bound to the UAUAUA motif and Nab2 bound to poly(A) at the 3′ end of mRNAs, in the context of a cleavage/polyadenylation complex, assist in correct mRNP maturation.

Functions for Nab2 in RNA surveillance are consistent with its interactions with Rrp6 and Trf4, and its role in intron-containing pre-mRNA turnover and Rrp6-dependent autoregulation of the *NAB2* transcript (Roth et al., 2009; Schmid et al., 2012). A function for Hrp1 in surveillance is consistent with reports that Hrp1 participates in Nrd1-dependent termination (Kuehner and Brow, 2008) and cytoplasmic nonsense-mediated decay (NMD) (González et al., 2000). The extensive contacts made by Hrp1 and Nab2 across the body of transcripts is consistent with ChIP data (González-Aguilera et al., 2011; Kim et al., 2004), whereas the human Hrp1 homolog TDP-43 is a ubiquitous RNP component with lncRNAs among its targets (Tollervey et al., 2011). These data suggest that Nab2, Hrp1, and TDP-43 function widely as RNA packaging factors. Nab2 is reported to fold poly(A) tails into a particular configuration (Viphakone et al., 2008), and in Nab2 mutants mRNAs are both hyperadenylated and aberrantly compacted (Brockmann et al., 2012). This suggests that RNA packaging and processing are tightly coupled, and appropriate RNA folding by Nab2 and Hrp1 might regulate access to the mRNA 3′ end and/or recruitment of surveillance and cleavage/polyadenylation factors. RNA fate may be determined not only by the protein factors bound but also by the folding of the RNA.

The ability of SUTs to undergo mRNA-like cleavage and polyadenylation may explain why SUTs are more stable than CUTs (Figure 6). However, unlike mRNAs, most SUTs are retained in the nucleus, with only a minority showing "mRNA-like" RNP compositions. We propose that additional layers of regulation following cleavage and polyadenylation

(D) Prevalence of tRNAs among all (blue) or A-tailed (red) RNA fragments recovered for Mtr4, Ski2, and Xrn1.
(E) Average distribution of all (gray) and A-tailed (blue) Ski2 and Mtr4 hits across scaled mRNAs.
(F) Distribution of Mtr4 hits across individual mRNAs aligned by their TSSs (n = 1,000).
(G) Average distribution of all (gray) and A-tailed (blue) Nab2 hits across scaled mRNAs (left), and average distribution of Nab2 hits with different length A-tails across scaled mRNAs (right).
(H) Average distribution around mRNA TSSs and 3′ ends of (1) Mtr4-, Nrd1-, and Nab3-binding sites (CRAC) (Wlotzka et al., 2011), (2) elongating Pol II (Churchman and Weissman, 2011) and (3) 3′ ends of unstable transcripts ("CUT fraction") (Neil et al., 2009).

determine whether a transcript is retained or exported. One candidate is Hek2, which bound to mRNAs and some "mRNA-like" SUTs but not to nuclear-restricted lncRNAs (Figure S2C). Hek2 has roles in mRNA stabilization and localization, and a human homolog (αCP2) enhances the 3′ end processing and stability of hα-mRNA (Ji et al., 2011). We suggest that Hek2 contributes to a decision point associated with 3′ end formation and selectively marks mRNAs, and some SUTs, for export. We predict that other sequence-specific binding proteins assist in the export of the "mRNA-like" SUTs that are not bound by Hek2.

In conclusion, our systematic analysis of RNP composition sheds light on how diverse classes of transcripts are distinguished in the cell and provides insights into the functions of these transcripts and of key RNP proteins.

EXPERIMENTAL PROCEDURES

Crosslinking and Analysis of cDNAs

We used the CRAC method as previously described (Granneman et al., 2011). In vivo protein:RNA crosslinks were generated by irradiating yeast cultures with UV light (254 nm, 100 s). Illumina sequencing reads were aligned to the yeast genome (SGD v64) using Novoalign. To quantify hits for particular genomic features and identify binding motifs, we used the pyCRAC package developed by Sander Granneman and custom Python/AWK scripts (available upon request). Analyses of hits in spliced versus unspliced transcripts and of non-genome-encoded oligo(A) tails were performed as previously described (Schneider et al., 2012; Wlotzka et al., 2011), and k-medians clustering used Cluster 3.0. Further information is provided in the extended experimental procedures and Table S5.

ACCESSION NUMBERS

The GEO accession number for all sequence data reported in this paper is GSE46742.

SUPPLEMENTAL INFORMATION

Supplemental Information includes Extended Experimental Procedures, two figures, and five tables and can be found with this article online at http://dx.doi.org/10.1016/j.cell.2013.07.047.

ACKNOWLEDGMENTS

We thank Sander Granneman and Grzegorz Kudla for help with analyses and Ola Helwak and Jai Tree for comments on the manuscript. This work was supported by Wellcome Trust grants (086587 and 077248) and European Commission 7th Framework Programme (UNICELLSYS, Grant No. 201142). Work in the Wellcome Trust Centre for Cell Biology is supported by Wellcome Trust core funding (092076). A.C.T. and D.T. designed the experiments; A.C.T. performed experiments and analyzed the data; A.C.T. and D.T. wrote the paper.

Received: May 7, 2013
Revised: June 25, 2013
Accepted: July 31, 2013
Published: August 29, 2013

REFERENCES

Batisse, J., Batisse, C., Budd, A., Böttcher, B., and Hurt, E. (2009). Purification of nuclear poly(A)-binding protein Nab2 reveals association with the yeast transcriptome and a messenger ribonucleoprotein core structure. J. Biol. Chem. *284*, 34911–34917.

Bousquet-Antonelli, C., Presutti, C., and Tollervey, D. (2000). Identification of a regulated pathway for nuclear pre-mRNA turnover. Cell *102*, 765–775.

Brar, G.A., Yassour, M., Friedman, N., Regev, A., Ingolia, N.T., and Weissman, J.S. (2012). High-resolution view of the yeast meiotic program revealed by ribosome profiling. Science *335*, 552–557.

Brockmann, C., Soucek, S., Kuhlmann, S.I., Mills-Lujan, K., Kelly, S.M., Yang, J.C., Iglesias, N., Stutz, F., Corbett, A.H., Neuhaus, D., and Stewart, M. (2012). Structural basis for polyadenosine-RNA binding by Nab2 Zn fingers and its function in mRNA nuclear export. Structure *20*, 1007–1018.

Camblong, J., Beyrouthy, N., Guffanti, E., Schlaepfer, G., Steinmetz, L.M., and Stutz, F. (2009). Trans-acting antisense RNAs mediate transcriptional gene cosuppression in S. cerevisiae. Genes Dev. *23*, 1534–1545.

Castelnuovo, M., Rahman, S., Guffanti, E., Infantino, V., Stutz, F., and Zenklusen, D. (2013). Bimodal expression of PHO84 is modulated by early termination of antisense transcription. Nat. Struct. Mol. Biol. *20*, 851–858.

Churchman, L.S., and Weissman, J.S. (2011). Nascent transcript sequencing visualizes transcription at nucleotide resolution. Nature *469*, 368–373.

Ciais, D., Bohnsack, M.T., and Tollervey, D. (2008). The mRNA encoding the yeast ARE-binding protein Cth2 is generated by a novel 3′ processing pathway. Nucleic Acids Res. *36*, 3075–3084.

Creamer, T.J., Darby, M.M., Jamonnak, N., Schaughency, P., Hao, H., Wheelan, S.J., and Corden, J.L. (2011). Transcriptome-wide binding sites for components of the Saccharomyces cerevisiae non-poly(A) termination pathway: Nrd1, Nab3, and Sen1. PLoS Genet. *7*, e1002329.

David, L., Huber, W., Granovskaia, M., Toedling, J., Palm, C.J., Bofkin, L., Jones, T., Davis, R.W., and Steinmetz, L.M. (2006). A high-resolution map of transcription in the yeast genome. Proc. Natl. Acad. Sci. USA *103*, 5320–5325.

Davis, C.A., and Ares, M., Jr. (2006). Accumulation of unstable promoter-associated transcripts upon loss of the nuclear exosome subunit Rrp6p in *Saccharomyces cerevisiae*. Proc. Natl. Acad. Sci. USA *103*, 3262–3267.

Faza, M.B., Chang, Y., Occhipinti, L., Kemmler, S., and Panse, V.G. (2012). Role of Mex67-Mtr2 in the nuclear export of 40S pre-ribosomes. PLoS Genet. *8*, e1002915.

Geisler, S., Lojek, L., Khalil, A.M., Baker, K.E., and Coller, J. (2012). Decapping of long noncoding RNAs regulates inducible genes. Mol. Cell *45*, 279–291.

González, C.I., Ruiz-Echevarría, M.J., Vasudevan, S., Henry, M.F., and Peltz, S.W. (2000). The yeast hnRNP-like protein Hrp1/Nab4 marks a transcript for nonsense-mediated mRNA decay. Mol. Cell *5*, 489–499.

González-Aguilera, C., Tous, C., Babiano, R., de la Cruz, J., Luna, R., and Aguilera, A. (2011). Nab2 functions in the metabolism of RNA driven by polymerases II and III. Mol. Biol. Cell *22*, 2729–2740.

Görnemann, J., Kotovic, K.M., Hujer, K., and Neugebauer, K.M. (2005). Cotranscriptional spliceosome assembly occurs in a stepwise fashion and requires the cap binding complex. Mol. Cell *19*, 53–63.

Granneman, S., Petfalski, E., and Tollervey, D. (2011). A cluster of ribosome synthesis factors regulate pre-rRNA folding and 5.8S rRNA maturation by the Rat1 exonuclease. EMBO J. *30*, 4006–4019.

Gudipati, R.K., Neil, H., Feuerbach, F., Malabat, C., and Jacquier, A. (2012a). The yeast RPL9B gene is regulated by modulation between two modes of transcription termination. EMBO J. *31*, 2427–2437.

Gudipati, R.K., Xu, Z., Lebreton, A., Séraphin, B., Steinmetz, L.M., Jacquier, A., and Libri, D. (2012b). Extensive degradation of RNA precursors by the exosome in wild-type cells. Mol. Cell *48*, 409–421.

Hasegawa, Y., Irie, K., and Gerber, A.P. (2008). Distinct roles for Khd1p in the localization and expression of bud-localized mRNAs in yeast. RNA *14*, 2333–2347.

Hieronymus, H., and Silver, P.A. (2003). Genome-wide analysis of RNA-protein interactions illustrates specificity of the mRNA export machinery. Nat. Genet. *33*, 155–161.

Hilleren, P.J., and Parker, R. (2003). Cytoplasmic degradation of splice-defective pre-mRNAs and intermediates. Mol. Cell *12*, 1453–1465.

Hu, W., Sweet, T.J., Chamnongpol, S., Baker, K.E., and Coller, J. (2009). Co-translational mRNA decay in Saccharomyces cerevisiae. Nature *461*, 225–229.

Iglesias, N., Tutucci, E., Gwizdek, C., Vinciguerra, P., Von Dach, E., Corbett, A.H., Dargemont, C., and Stutz, F. (2010). Ubiquitin-mediated mRNP dynamics and surveillance prior to budding yeast mRNA export. Genes Dev. *24*, 1927–1938.

Irie, K., Tadauchi, T., Takizawa, P.A., Vale, R.D., Matsumoto, K., and Herskowitz, I. (2002). The Khd1 protein, which has three KH RNA-binding motifs, is required for proper localization of ASH1 mRNA in yeast. EMBO J. *21*, 1158–1167.

Ji, X., Kong, J., and Liebhaber, S.A. (2011). An RNA-protein complex links enhanced nuclear 3′ processing with cytoplasmic mRNA stabilization. EMBO J. *30*, 2622–2633.

Kessler, M.M., Henry, M.F., Shen, E., Zhao, J., Gross, S., Silver, P.A., and Moore, C.L. (1997). Hrp1, a sequence-specific RNA-binding protein that shuttles between the nucleus and the cytoplasm, is required for mRNA 3′-end formation in yeast. Genes Dev. *11*, 2545–2556.

Kim, M., Ahn, S.-H., Krogan, N.J., Greenblatt, J.F., and Buratowski, S. (2004). Transitions in RNA polymerase II elongation complexes at the 3′ ends of genes. EMBO J. *23*, 354–364.

Kim, M., Vasiljeva, L., Rando, O.J., Zhelkovsky, A., Moore, C., and Buratowski, S. (2006). Distinct pathways for snoRNA and mRNA termination. Mol. Cell *24*, 723–734.

Kim Guisbert, K., Duncan, K., Li, H., and Guthrie, C. (2005). Functional specificity of shuttling hnRNPs revealed by genome-wide analysis of their RNA binding profiles. RNA *11*, 383–393.

Kuehner, J.N., and Brow, D.A. (2008). Regulation of a eukaryotic gene by GTP-dependent start site selection and transcription attenuation. Mol. Cell *31*, 201–211.

Larochelle, M., Lemay, J.-F., and Bachand, F. (2012). The THO complex cooperates with the nuclear RNA surveillance machinery to control small nucleolar RNA expression. Nucleic Acids Res. *40*, 10240–10253.

Lund, M.K., and Guthrie, C. (2005). The DEAD-box protein Dbp5p is required to dissociate Mex67p from exported mRNPs at the nuclear rim. Mol. Cell *20*, 645–651.

Mauchi, N., Ohtake, Y., and Irie, K. (2010). Stability control of MTL1 mRNA by the RNA-binding protein Khd1p in yeast. Cell Struct. Funct. *35*, 95–105.

Mayer, A., Lidschreiber, M., Siebert, M., Leike, K., Söding, J., and Cramer, P. (2010). Uniform transitions of the general RNA polymerase II transcription complex. Nat. Struct. Mol. Biol. *17*, 1272–1278.

Mayer, A., Heidemann, M., Lidschreiber, M., Schreieck, A., Sun, M., Hintermair, C., Kremmer, E., Eick, D., and Cramer, P. (2012). CTD tyrosine phosphorylation impairs termination factor recruitment to RNA polymerase II. Science *336*, 1723–1725.

Mitchell, S.F., Jain, S., She, M., and Parker, R. (2013). Global analysis of yeast mRNPs. Nat. Struct. Mol. Biol. *20*, 127–133.

Neil, H., Malabat, C., d'Aubenton-Carafa, Y., Xu, Z., Steinmetz, L.M., and Jacquier, A. (2009). Widespread bidirectional promoters are the major source of cryptic transcripts in yeast. Nature *457*, 1038–1042.

Paquin, N., and Chartrand, P. (2008). Local regulation of mRNA translation: new insights from the bud. Trends Cell Biol. *18*, 105–111.

Parker, R. (2012). RNA degradation in Saccharomyces cerevisae. Genetics *191*, 671–702.

Pelechano, V., Wei, W., and Steinmetz, L.M. (2013). Extensive transcriptional heterogeneity revealed by isoform profiling. Nature *497*, 127–131.

Pérez-Cañadillas, J.M. (2006). Grabbing the message: structural basis of mRNA 3'UTR recognition by Hrp1. EMBO J. *25*, 3167–3178.

Porrua, O., and Libri, D. (2013). RNA quality control in the nucleus: the Angels' share of RNA. Biochim. Biophys. Acta *1829*, 604–611.

Reis, C.C., and Campbell, J.L. (2007). Contribution of Trf4/5 and the nuclear exosome to genome stability through regulation of histone mRNA levels in *Saccharomyces cerevisiae*. Genetics *175*, 993–1010.

Rhee, H.S., and Pugh, B.F. (2012). Genome-wide structure and organization of eukaryotic pre-initiation complexes. Nature *483*, 295–301.

Rondón, A.G., Jimeno, S., and Aguilera, A. (2010). The interface between transcription and mRNP export: from THO to THSC/TREX-2. Biochim. Biophys. Acta *1799*, 533–538.

Roth, K.M., Byam, J., Fang, F., and Butler, J.S. (2009). Regulation of NAB2 mRNA 3′-end formation requires the core exosome and the Trf4p component of the TRAMP complex. RNA *15*, 1045–1058.

Schmid, M., Poulsen, M.B., Olszewski, P., Pelechano, V., Saguez, C., Gupta, I., Steinmetz, L.M., Moore, C., and Jensen, T.H. (2012). Rrp6p controls mRNA poly(A) tail length and its decoration with poly(A) binding proteins. Mol. Cell *47*, 267–280.

Schneider, C., Kudla, G., Wlotzka, W., Tuck, A.C., and Tollervey, D. (2012). Transcriptome-wide analysis of exosome targets. Mol. Cell *48*, 422–433.

Schwer, B., Erdjument-Bromage, H., and Shuman, S. (2011). Composition of yeast snRNPs and snoRNPs in the absence of trimethylguanosine caps reveals nuclear cap binding protein as a gained U1 component implicated in the cold-sensitivity of tgs1Δ cells. Nucleic Acids Res. *39*, 6715–6728.

Thiebaut, M., Colin, J., Neil, H., Jacquier, A., Séraphin, B., Lacroute, F., and Libri, D. (2008). Futile cycle of transcription initiation and termination modulates the response to nucleotide shortage in S. cerevisiae. Mol. Cell *31*, 671–682.

Tollervey, J.R., Curk, T., Rogelj, B., Briese, M., Cereda, M., Kayikci, M., König, J., Hortobágyi, T., Nishimura, A.L., Zupunski, V., et al. (2011). Characterizing the RNA targets and position-dependent splicing regulation by TDP-43. Nat. Neurosci. *14*, 452–458.

Tran, E.J., Zhou, Y., Corbett, A.H., and Wente, S.R. (2007). The DEAD-box protein Dbp5 controls mRNA export by triggering specific RNA:protein remodeling events. Mol. Cell *28*, 850–859.

van Dijk, E.L., Chen, C.L., d'Aubenton-Carafa, Y., Gourvennec, S., Kwapisz, M., Roche, V., Bertrand, C., Silvain, M., Legoix-Né, P., Loeillet, S., et al. (2011). XUTs are a class of Xrn1-sensitive antisense regulatory non-coding RNA in yeast. Nature *475*, 114–117.

Vasiljeva, L., and Buratowski, S. (2006). Nrd1 interacts with the nuclear exosome for 3′ processing of RNA polymerase II transcripts. Mol. Cell *21*, 239–248.

Viphakone, N., Voisinet-Hakil, F., and Minvielle-Sebastia, L. (2008). Molecular dissection of mRNA poly(A) tail length control in yeast. Nucleic Acids Res. *36*, 2418–2433.

Vogel, C., Silva, G.M., and Marcotte, E.M. (2011). Protein expression regulation under oxidative stress. Mol. Cell. Proteomics *10*, M111, 009217.

Wagschal, A., Rousset, E., Basavarajaiah, P., Contreras, X., Harwig, A., Laurent-Chabalier, S., Nakamura, M., Chen, X., Zhang, K., Meziane, O., et al. (2012). Microprocessor, Setx, Xrn2, and Rrp6 co-operate to induce premature termination of transcription by RNAPII. Cell *150*, 1147–1157.

Wlotzka, W., Kudla, G., Granneman, S., and Tollervey, D. (2011). The nuclear RNA polymerase II surveillance system targets polymerase III transcripts. EMBO J. *30*, 1790–1803.

Wolf, J.J., Dowell, R.D., Mahony, S., Rabani, M., Gifford, D.K., and Fink, G.R. (2010). Feed-forward regulation of a cell fate determinant by an RNA-binding protein generates asymmetry in yeast. Genetics *185*, 513–522.

Wong, C.M., Qiu, H., Hu, C., Dong, J., and Hinnebusch, A.G. (2007). Yeast cap binding complex impedes recruitment of cleavage factor IA to weak termination sites. Mol. Cell. Biol. *27*, 6520–6531.

Xu, Z., Wei, W., Gagneur, J., Perocchi, F., Clauder-Münster, S., Camblong, J., Guffanti, E., Stutz, F., Huber, W., and Steinmetz, L.M. (2009). Bidirectional promoters generate pervasive transcription in yeast. Nature *457*, 1033–1037.

EXTENDED EXPERIMENTAL PROCEDURES

Strains and Media

S. cerevisiae strains with genomically encoded tagged proteins were generated by standard methods and grown at 30°C to A_{600} ~ 0.5. CRAC strains expressed C-terminally HTP-tagged (His_6-TEV-Protein A) proteins under the control of their endogenous promoter and were grown in synthetic dropout media with glucose. Depletion strains were grown in rich media with galactose, then cultures supplemented with 2% glucose to repress expression of N-terminally HA_3-tagged Hrp1 or Nab2 under the control of the *GAL1* promoter. Protein depletion was verified by western blotting. Detailed strain information is provided in Table S5.

Plasmids and Oligonucleotides

The plasmids and oligonucleotides used in this study are described in Table S5.

Crosslinking and Analyses of cDNAs (CRAC)

We used the CRAC technique largely as previously described (Granneman et al., 2009; Granneman et al., 2011), but include full details here:

Cell pellets were vortexed with 1 ml TN150 (50 mM Tris-HCl [pH 7.8], 150 mM NaCl, 0.1% (v/v) NP-40, 5 mM β-mercaptoethanol, EDTA-free protease inhibitor cocktail [Roche]) and 2.5 ml zirconia beads (Thistle Scientific) for 5x 1 min pulses, cooling on ice in between. Cell lysates were diluted with an additional 3 ml TN150, and debris removed by centrifugation (20 min, 4,600 × *g*; then 20 min, 20,000 × *g*; 4°C). Cleared lysates were incubated with 125 μl IgG beads (IgG Sepharose 6 Fast Flow, GE), rotating at 4°C for 2 hr. Beads were washed with TN150 (2× 10 ml) then TN1000 (2× 10 ml; as TN150, but with 1 M NaCl), then His-tagged RNA:protein complexes eluted by TEV cleavage in TN150 (1.5 μl homemade GST-TEV, 2 hr, 18°C). The eluate was treated with RNace-IT (Agilent; 0.1 units, 5 min, 37°C) to fragment protein-bound RNA, and added to 400 mg guanidine-HCl to quench RNase activity. The solution was adjusted for nickel affinity purification by the addition of 27 μl NaCl (5.0 M) and 3 ul imidazole (2.5 M), and added to 50 ul nickel beads (Ni-NTA agarose, QIAGEN). After an overnight incubation (4°C), the nickel beads were transferred to a spin column (Snap Cap, Pierce) and washed three times with WBI (50 mM Tris-HCl [pH 7.8], 300 mM NaCl, 0.1% NP-40, 10 mM imidazole, 5 mM β-mercaptoethanol, 6.0 M guanidine-HCl) then three times with 1xPNK (50 mM Tris-HCl [pH 7.8], 10 mM $MgCl_2$, 0.5% NP-40, 5 mM β-mercaptoethanol). Several on-bead reactions (total volume 80 μl in each case) were then performed, washing once with WBI and three times with 1xPNK after each reaction:

1. TSAP (Promega) phosphatase treatment – 30 min, 37°C, in 1xPNK.
2. Preadenylated 3′ miRCat-33 linker (IDT) ligation using T4 RNA ligase (NEB) – 6 hr, 25°C, in 1xPNK.
3. 5′ end labeling with [γ^{32}P]-ATP using T4 polynucleotide kinase (Sigma) – 1 hr, 37°C, in 1xPNK, with addition of 100 nmol ATP after 40 min.
4. 5′ linker ligation using T4 RNA ligase (NEB) – 16 hr, 16°C, in 1xPNK.

The beads were then washed three times with WBII (50 mM Tris-HCl [pH 7.8], 50 mM NaCl, 0.1% (v/v) NP-40, 10 mM imidazole, 5 mM β-mercaptoethanol), and RNA:protein complexes eluted into EB (50 mM Tris-HCl [pH 7.8], 50 mM NaCl, 0.1% NP-40, 150 mM imidazole, 5 mM β-mercaptoethanol) and precipitated with TCA (20% (v/v) final concentration). After washing with acetone, pellets were resuspended in NuPAGE 1x LDS sample loading buffer (Invitrogen) and protein:RNA complexes resolved by electrophoresis (4%–12% Bis-Tris NuPAGE gel, Invitrogen; 150 V). After electrophoretic transfer to a Hybond C nitrocellulose membrane (GE) in 1x NuPAGE transfer buffer (1.5 hr, 100V; Invitrogen), labeled RNA was detected by autoradiography. The appropriate regions were excised from the membrane, and treated with Proteinase K (Roche) in WBII containing 1% (w/v) SDS and 5 mM EDTA to release RNA (55°C, 2 hr). RNA was isolated by phenol:chloroform extraction followed by ethanol precipitation, resuspending in 11 μl water.

The RNA was reverse transcribed with Superscript III (Invitrogen; 1 hr, 50°C), using the miRCat-33 RT oligo (IDT). After heat inactivation (15 min, 65°C), samples were treated with RNase H (NEB; 30 min, 37°C). The cDNA was amplified by PCR using LA Taq (Takara; 19–24 cycles, 52°C annealing temperature). PCR products were precipitated using ethanol, resuspended in 1x gel loading dye (NEB) and resolved on a 3% Metaphor agarose gel (Lonza). A region corresponding to ~120–300 bp was excised from each lane, and DNA extracted using a QIAGEN gel purification kit, eluting in 20 μl water.

The libraries were checked by Sanger sequencing. Briefly, 2 μl of the purified PCR product was cloned into a pCR4 TOPO vector and transformed into TOP10 cells (Invitrogen) according to manufacturer's instructions. Colonies were picked, inoculated into LB medium with ampicillin, grown overnight at 30°C, and plasmid DNA extracted using a Plasmid Mini kit (QIAGEN). Sequencing reactions were performed using the Big Dye kit (Applied Biosystems) and the M13 F primer supplied with the pCR4 TOPO vector (Invitrogen).

For Solexa sequencing, libraries were sent to Genepool (University of Edinburgh) or Source Bioscience.

Northern Analyses

RNA was extracted by hot phenol extraction, northern hybridizations with riboprobes performed using Ultrahyb (Ambion), and signals detected using a Fuji FLA-5100 PhosphorImager or by autoradiography.

Bioinformatic Analyses

Quality Filtering and Read Mapping

Raw data were preprocessed using the fastx toolkit, specifically the fastx_clipper to remove 3′ sequencing adapters, fastq_quality_trimmer to trim low-quality positions from the 3′ end of reads, fastq_quality_filter to remove reads without a high-quality score throughout, and fastx_artifacts_filter to remove homopolymeric sequencing artifacts. Most of the 5′ linkers used to prepare CRAC libraries contain a random 3 nt sequence, which enabled PCR duplicates (reads amplified from a single cDNA) to be removed by collapsing identical sequences. The 5′ linkers also contain a barcode, enabling samples to be multiplexed for sequencing. Following preprocessing, we separated reads by barcode then mapped them to the yeast genome (SGD v64) using Novoalign, and we refer to mapped reads as "hits." To remove PCR duplicates that were not collapsed during preprocessing due to sequencing errors or differential trimming at the 3′ end by fastx_quality_trimmer, we collapsed any reads with the same random 3 nt tag in their 5′ linker and with 5′ ends mapping to the same genomic coordinate.

We downloaded gene annotations from Ensembl (EF4.68), and supplemented them with the coordinates of UTRs, CUTs and SUTs (Xu et al., 2009), additional antisense and intergenic lncRNAs (Granovskaia et al., 2010; Yassour et al., 2010) and Xrn1-sensitive unstable transcripts (van Dijk et al., 2011). To count hits for each genomic feature we used the pyCRAC package developed by Sander Granneman (source code and documentation available from https://bitbucket.org/sgrann/pycrac). Briefly, mapped reads from the Novoalign output file are corrected for the presence of insertions, deletions or substitutions, then the corrected reads overlapping each genomic feature counted. We included flanking regions around mRNAs of up to 50 nt at the 5′ end and 300 nt at the 3′ end to catch hits falling outside of misannotated features. This produced a "hit table" for each sample. To assess the similarity between samples we calculated the Spearman rank correlation coefficient between pairs of hit tables, considering all mRNAs, CUTs, SUTs, tRNAs, snRNAs, snoRNAs and rRNAs detected in at least two data sets.

Plots of Hit Distributions across Genomic Features

To examine the distribution of hits across the length of individual genomic features we used the pyCRAC package to count the number of mapped reads overlapping each nucleotide along the feature of interest. To examine the distribution of hits for a particular protein across all members of a transcript class (e.g., Cbc1 hits in mRNAs), we used two related approaches, both performed on the most abundantly bound members of the transcript class.

In the first approach, which provides an average binding profile across all transcripts, each transcript was divided into 100 bins of equal length, and 100 nt 5′ and 3′ flanking regions divided into 10 bins (120 bins in total). Considering the first transcript, hits were counted for each nucleotide, then divided by the length of each bin to obtain hit densities. This was repeated for 1,000 transcripts, then each transcript normalized by linear scaling so that the densities for that transcript summed to 100. We then averaged the 1,000 individual profiles, and the resulting plot reflects the typical hit distribution across all 1,000 transcripts, with the normalization step ensuring that each transcript contributes equally.

We complemented this approach with an analysis in which 1,000 transcripts were sorted by length, hits counted at each position and scaled to the maximum value for each transcript, and the data plotted as a two dimensional heat map (Figure 5F). Here, each row represents a transcript, and each column the absolute position from the aligned TSSs. This enables the individual hit distributions of 1,000 transcripts to be displayed on one plot, without scaling by length. We used a similar approach to plot the distribution of CRAC hits or other transcriptome-wide data in 300-900 nt windows aligned to mRNA transcription start and poly(A) sites (Figure 5H), but in this case plotted the average rather than individual distributions.

Motif Analyses

To search for sequence motifs, we used the pyCRAC package to calculate statistical overrepresentation scores for each possible k-mer according to the previously described algorithm (Wlotzka et al., 2011). High Z scores indicate that a motif is significantly more abundant within hits than would be expected by chance, taking into account the sequence composition of the transcripts to which the hits map. To avoid detecting spurious motifs arising from sequencing artifacts or adapters that were not removed, we only used reads for which the 3′ adaptor was detected. To restrict the analysis to encoded motifs, we used the genomic sequence corresponding to each mapped read. We also excluded low-complexity reads (with fewer than 7 nonmodal nucleotides, e.g., "GTCCGAAAAAAAAA" would be excluded) to avoid the artifactual detection of oligo(A) motifs for reads with short non-genome-encoded oligo(A) tails that can map to A-rich regions of the genome. Having identified a motif, we then plotted the distribution of hits and deletions around all occurrences of that motif in the transcriptome using the pyCRAC package. Using a Novoalign file as input, this counts the number of mapped reads overlapping each nucleotide (e.g., −100 nt to +100 nt) around all occurrences of a given motif, then sums the scores for each nucleotide. We used a similar strategy to plot hits around stop codons.

To examine the presence of different length $(CNN)_n$ repeats in different transcript classes, we counted the number of transcripts in which the longest CNN repeat was $(CNN)_1$, $(CNN)_2$, $(CNN)_3$, … $(CNN)_n$. We then plotted the proportion of transcripts for each value of n.

Pre-mRNA Analyses

To identify hits in spliced mRNAs versus unspliced pre-mRNAs we mapped reads to a library of spliced transcripts and another of unspliced transcripts, as previously described (Schneider et al., 2012). We considered only reads mapping to intron-containing genes, and calculated the ratio of hits across exon-exon junctions to hits across intron-exon junctions.

Clustering Analysis

To classify transcripts by their "RNP profiles," we extracted the number of hits for each mRNA, CUT and SUT from Cbc1, Mtr4, Nab2, Mex67, Ski2 and Xrn1 hit tables. We included replicate data sets, so there were 18 data sets in total. Each data set was then

normalized to hits per million hits in mRNAs, CUTs and SUTs, and transcripts rejected if they did not have at least 50 hits per million in two data sets. To remove transcripts for which data were not reproducible, we rejected transcripts with Spearman rank correlation coefficients of < 0.37 when comparing two replicate sets of observations for the 6 proteins tested. For each transcript, we then averaged replicate observations (mean) to reduce the influence of experimental variation upon the clustering analysis. To account for differences in transcript abundance, we normalized the data for each gene ($\Sigma i^2 = 1$). This produced a set of 4,960 RNP profiles, which reflect the relative binding of a transcript to each of the six tested proteins.

We also included Pab1 in our analysis, but instead of total hits used a measure of peak sharpness to distinguish bona fide Pab1 interactions at the 3′ end of transcripts (sharp peak) versus nonspecific interactions across transcript bodies (broad distribution). Briefly, for each transcript the nucleotide with the greatest number of Pab1 hits was identified, and the height of this binding peak divided by the maximum height when reads in a 400 nt window centered on this peak were placed randomly. Scores were scaled to occupy a range from 0 to 1. Transcripts were then clustered by their RNP profiles (including the Pab1 score) using Cluster 3.0 (k-medians, k = 10, Euclidean distance). The data were displayed as a heat map, with all replicates (rather than averages across two or three replicates) shown. The third replicate data set for Cbc1, Mtr4, Nab2 and Mex67 was not used in the initial Spearman rank filtering step, enabling us to verify that the data had not been over-fitted.

For proteins not included in the clustering analysis (e.g., Hek2, Tho2 and Gbp2), the amount of binding to transcripts in each cluster (or a set of clusters) was examined using box-and-whisker plots to summarize the distribution of transcript hit totals (raw hit numbers). The stabilization of mRNAs or SUTs in surveillance mutants (Gudipati et al., 2012) was examined for each cluster by plotting cumulative frequency distributions, as described in (Gudipati et al., 2012). Binding to ribosomes was examined using ribosome profiling data (Brar et al., 2012) (GEO sample GSM843748). Briefly, the first 25 nt was extracted from each raw read, mapped to the yeast genome using Novoalign, and the number of reads for each annotated mRNA or SUT counted using pyCRAC. The raw number of hits are presented.

GO term analyses were performed using the SGD GO Term Finder (http://www.yeastgenome.org).

Analyses of Non-Genome-Encoded oligo(A) Tails

To identify reads with non-genome-encoded oligo(A) tails, we employed a pipeline developed by Grzegorz Kudla (Wlotzka et al., 2011). We first selected reads where the 3′ adaptor was identified and clipped in the data preprocessing steps. We then used blastall to identify the region of the read mapping to the yeast genome. Where the mapped region did not extend to the 3′ end of the clipped read, the remaining nucleotides were classed as non-genome-encoded. We selected reads where the non-genome-encoded portion contained two or more As, and fewer than one in five non-A residues. We classed these reads as having non-genome-encoded oligo(A) tails. We then analyzed these reads in the same way described for total reads, to identify which transcripts they mapped to, where within transcripts they mapped, and the proportion of A-tailed reads in different transcript classes. The RNA fragmentation step in the CRAC protocol preserves the length of A-tails, as RNase A and T1 only rarely cut after A residues, enabling the length distribution of A-tails to be analyzed for each sample. However, one limitation of this approach is that the adaptor, barcode and mapped portion of the read occupies ~31 nt, and so long A-tails (>~15 nt) are underrepresented. To address this limitation, we removed the requirement for reads to contain a mapped region, and instead simply counted the number of A residues at the 3′ end of each read. To avoid biases from different read length distributions in different data sets, we restricted this latter analysis to reads between 30 and 35 nt long (after removal of the 3′ adaptor).

Definition of Transcript 3′ Ends

To precisely locate sites of 3′ end formation on mRNAs and SUTs, we selected all reads in the Pab1 data set with non-genome-encoded oligo(A) tails, and extracted the genomic coordinate of the last genome-encoded nucleotide. We defined these as poly(A) (pA) sites, and for each gene selected the most frequently identified pA site (“major pA site”). To exclude adenosine-rich reads mapping with low confidence to genome-encoded oligo(A) tracts, we removed low-complexity reads (which we defined as reads with genome-encoded portions containing fewer than 8 nonmodal nucleotides). We also removed reads mapping within 200 nt of a TSS, as these may correspond to upstream transcripts. We then plotted the frequency of motifs (e.g., UAUAUA) or individual nucleotide bases (i.e., A, U, G or C) across the genomic region flanking each major pA site. We also used DREME to identify sequence motifs enriched in these regions.

SUPPLEMENTAL REFERENCES

Brachmann, C.B., Davies, A., Cost, G.J., Caputo, E., Li, J., Hieter, P., and Boeke, J.D. (1998). Designer deletion strains derived from *Saccharomyces cerevisiae* S288C: a useful set of strains and plasmids for PCR-mediated gene disruption and other applications. Yeast *14*, 115–132.

Granneman, S., Kudla, G., Petfalski, E., and Tollervey, D. (2009). Identification of protein binding sites on U3 snoRNA and pre-rRNA by UV cross-linking and high-throughput analysis of cDNAs. Proc. Natl. Acad. Sci. USA *106*, 9613–9618.

Granovskaia, M.V., Jensen, L.J., Ritchie, M.E., Toedling, J., Ning, Y., Bork, P., Huber, W., and Steinmetz, L.M. (2010). High-resolution transcription atlas of the mitotic cell cycle in budding yeast. Genome Biol. *11*, R24.

Gudipati, R.K., Xu, Z., Lebreton, A., Séraphin, B., Steinmetz, L.M., Jacquier, A., and Libri, D. (2012). Extensive degradation of RNA precursors by the exosome in wild-type cells. Mol. Cell *48*, 409–421.

Hentges, P., Van Driessche, B., Tafforeau, L., Vandenhaute, J., and Carr, A.M. (2005). Three novel antibiotic marker cassettes for gene disruption and marker switching in Schizosaccharomyces pombe. Yeast *22*, 1013–1019.

Longtine, M.S., McKenzie, A., 3rd, Demarini, D.J., Shah, N.G., Wach, A., Brachat, A., Philippsen, P., and Pringle, J.R. (1998). Additional modules for versatile and economical PCR-based gene deletion and modification in Saccharomyces cerevisiae. Yeast *14*, 953–961.

Swiatkowska, A., Wlotzka, W., Tuck, A., Barrass, J.D., Beggs, J.D., and Tollervey, D. (2012). Kinetic analysis of pre-ribosome structure in vivo. RNA *18*, 2187–2200.

Yassour, M., Pfiffner, J., Levin, J.Z., Adiconis, X., Gnirke, A., Nusbaum, C., Thompson, D.A., Friedman, N., and Regev, A. (2010). Strand-specific RNA sequencing reveals extensive regulated long antisense transcripts that are conserved across yeast species. Genome Biol. *11*, R87.

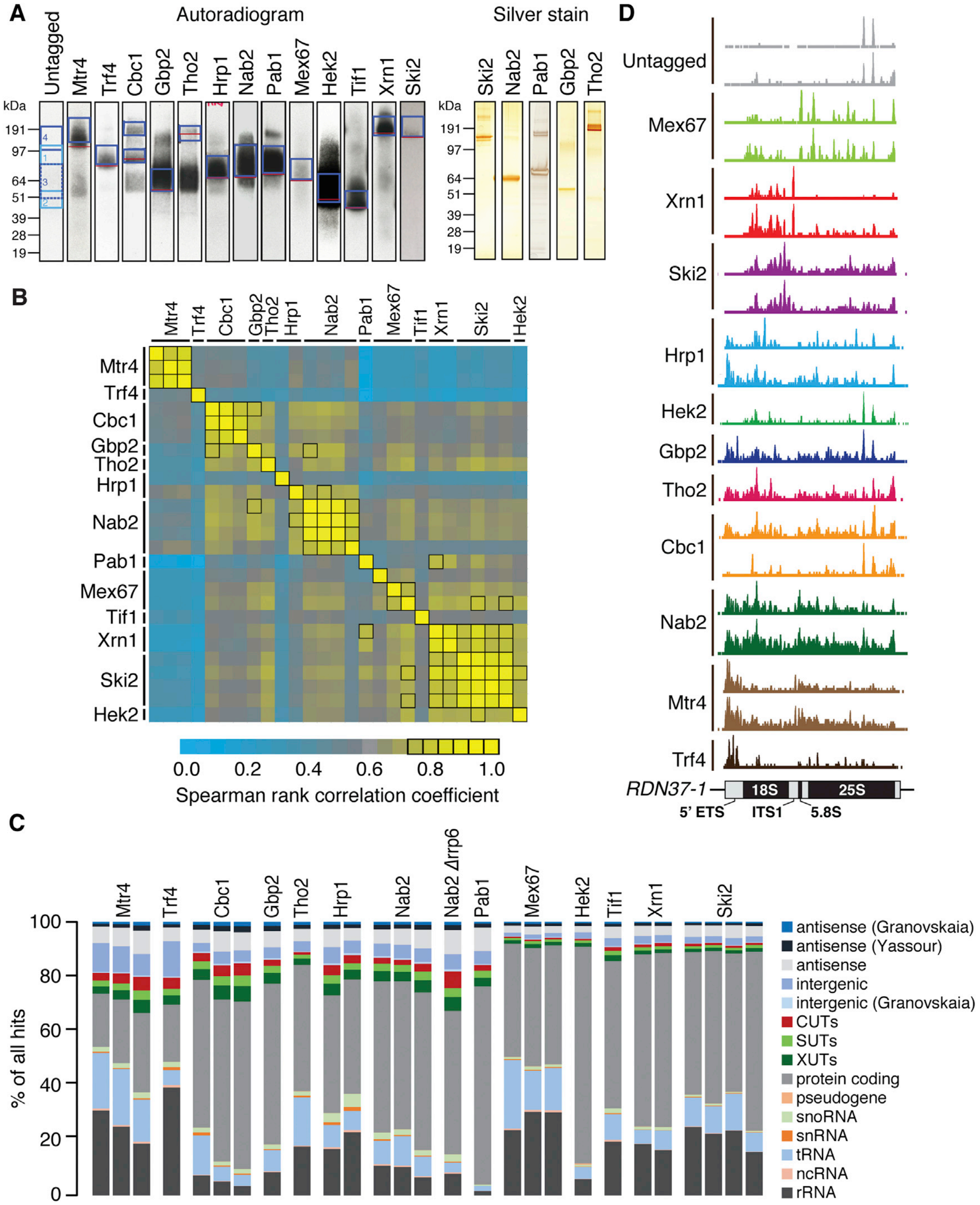

Figure S1. Transcriptome-wide Identification of Targets of Key RNA Maturation and Turnover Proteins, Related to Figure 1

(A) Left: autoradiogram of protein:RNA complexes purified by SDS-PAGE. Red bars indicate the migration of the proteins when analyzed by western blotting, and this information was used to select the appropriate regions (blue boxes) for excision. RNA fragments were then isolated from these excised regions and analyzed by high-throughput sequencing. Right: silver stain analysis of representative protein:RNA complexes following affinity purification.

(B) Pairwise Spearman rank comparison of CRAC data sets, based upon the number of hits for each protein in each annotated transcript.

(C) Breakdown of recovered RNA fragments by transcript class. We include several published sets of lncRNA annotations: CUTs (cryptic unstable transcripts) and SUTs (stable unannotated transcripts) (Xu et al., 2009), XUTs (Xrn1-sensitive unstable transcripts) (van Dijk et al., 2011), antisense lncRNAs (Yassour et al., 2010), and antisense/intergenic lncRNAs (Granovskaia et al., 2010). We also include our own antisense/intergenic annotations for transcripts not annotated in (Xu et al., 2009): transcripts mapping to genomic regions with no annotated feature on either strand are classed as "intergenic," and transcripts mapping to the strand opposite to an annotated feature are classed as "antisense."

(D) Distribution of binding sites across the 35S pre-rRNA, scaled to the maximum height for each data set.

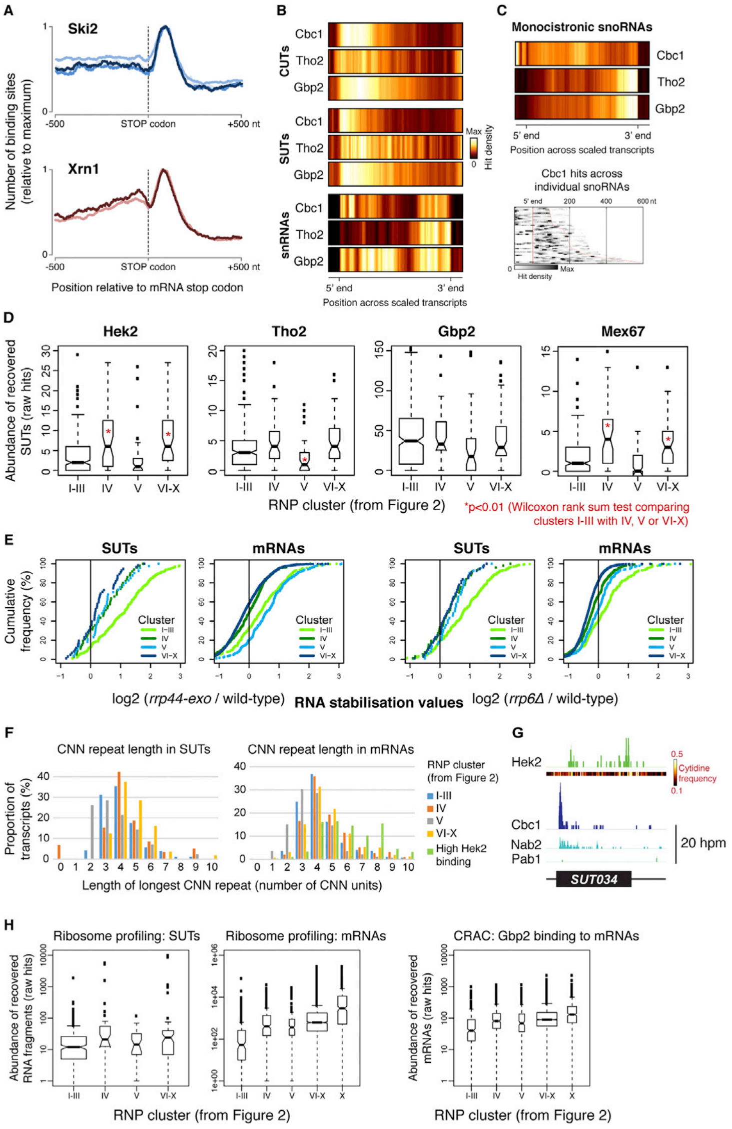

Figure S2. Functions of RNA-Binding Proteins in the Biogenesis and Turnover of mRNAs and lncRNAs, Related to Figure 2

(A) Distribution of Ski2- and Xrn1-binding sites around all mRNA stop codons. Overlapping reads are flattened into single contigs to avoid bias toward abundant transcripts.

(B) Average binding distribution of the cap-binding complex subunit Cbc1 and TREX components Gbp2 and Tho2 across CUTs, SUTs and snRNAs. Average hit densities were calculated for 120 bins spanning the 500 most abundantly bound CUTs or SUTs, and all snRNAs (including 2x10 bins for 100 nt flanking regions).

(C) Top: average binding distribution of Cbc1, Gbp2 and Tho2 across all monocistronic snoRNAs. Average hit densities were calculated for 120 bins (including 2x10 bins for 100 nt flanking regions). Bottom: Cbc1 hits across individual snoRNAs (distance in nt from the 5′ end of the mature snoRNA).

(D) Box and whisker plot of the number of hits for each SUT recovered bound to Hek2, Tho2, Gbp2 and Mex67. Transcripts are grouped by RNP profiles (see Figure 2). Asterisks indicate where median values are significantly different from the cluster I-III median (Wilcoxon rank sum test, $p < 0.01$).

(E) Cumulative distribution of log_2 stabilization ratios for SUTs and mRNAs in the indicated surveillance mutants (Gudipati et al., 2012). Transcripts are grouped by RNP profiles (Figure 2).

(F) Histogram showing the prevalence of SUTs (left) or mRNAs (right) with different length $(CNN)_n$ motifs. For each transcript, the longest CNN repeat was recorded, and the proportion of transcripts with each CNN repeat length plotted. Transcripts were divided into the clusters defined in Figure 2. For mRNAs, an additional category is included (green), comprising mRNAs with particularly high binding to Hek2 (determined by clustering analysis as per Figure 2, but with Hek2 included).

(G) Distribution of Hek2, Cbc1, Nab2 and Pab1 CRAC hits across SUT034. Cytidine frequency within the SUT sequence is indicated.

(H) Box and whisker plot of the total number of hits for each SUT or mRNA recovered in ribosome profiling experiments (Brar et al., 2012) (GEO sample GSM843748). Transcripts are divided into the clusters defined in Figure 2. Although cluster X mRNAs are included in the cluster VI-X category, we also show them separately as they appear to be particularly highly translated. For comparison, the number of Gbp2 CRAC hits for each mRNA is shown (right). The upper and lower edges of the boxes are the upper and lower quartiles, respectively. The whiskers extend from these edges to the most extreme value within 1.5 times the length of the box.
